# Phenotypic evolution through variation in splicing of the noncoding RNA *COOLAIR*

**DOI:** 10.1101/gad.258814.115

**Published:** 2015-04-01

**Authors:** Peijin Li, Zhen Tao, Caroline Dean

**Affiliations:** John Innes Centre, Norwich NR4 7UH, United Kingdom

**Keywords:** *FLOWERING LOCUS C*, *COOLAIR*, alternative splicing, flowering time, RNA capping/decapping

## Abstract

The extent to which natural polymorphisms in noncoding sequences have functional consequences is still unknown. A large proportion of the natural variation in flowering in *Arabidopsis thaliana* accessions is due to noncoding *cis* polymorphisms that define distinct haplotypes of *FLOWERING LOCUS C* (*FLC*). Here, we show that a single natural intronic polymorphism in one haplotype affects *FLC* expression and thus flowering by specifically changing splicing of the *FLC* antisense transcript *COOLAIR*. Altered antisense splicing increases *FLC* expression via a cotranscriptional mechanism involving capping of the *FLC* nascent transcript. Single noncoding polymorphisms can therefore be a major contributor to phenotypic evolution through modulation of noncoding transcripts.

## Abstract (precis)

A large proportion of the natural variation in flowering in *Arabidopsis thaliana* accessions is due to noncoding *cis* polymorphisms that define distinct haplotypes of *FLOWERING LOCUS C* (*FLC*). Li et al. find that a single natural intronic polymorphism in one haplotype affects *FLC* expression and thus flowering by specifically changing splicing of the *FLC* antisense transcript *COOLAIR*. Altered antisense splicing increases *FLC* expression via a cotranscriptional mechanism involving capping of the *FLC* nascent transcript.

Understanding the molecular basis of natural phenotypic change is a central question in biology (Alonso-Blanco and Mendez-Vigo 2014). We are studying the variation underpinning the adaptive evolution of *Arabidopsis thaliana* accessions focusing on flowering time, a trait with considerable fitness consequences (Dittmar et al. 2014). A large proportion of the natural variation in flowering maps to the floral repressor locus *FLOWERING LOCUS C* (*FLC*) (Shindo et al. 2006). Functionally distinct *FLC* haplotypes exist in the worldwide population, distinguished by polymorphisms in noncoding regions that influence expression level and the rate of epigenetic silencing of the gene (Li et al. 2014).

*FLC* regulation involves pathways that link different aspects of chromatin regulation with a set of antisense transcripts collectively called *COOLAIR* (Swiezewski et al. 2009; Liu et al. 2010; Castaings et al. 2014; Marquardt et al. 2014). Different *FLC* expression states are linked to alternative splicing and alternative polyadenylation of *COOLAIR* (Liu et al. 2007, 2010). Low *FLC* expression is associated with use of a small intron and polyadenylation at a proximal region, so-called class I *COOLAIR* variants (Liu et al. 2007; Hornyik et al. 2010; Marquardt et al. 2014; Wang et al. 2014). High *FLC* expression is associated with use of a large intron and polyadenylation at a distal region, the class II variants (Fig. 1). Cold exposure also influences *COOLAIR* splicing and polyadenylation (Swiezewski et al. 2009). This sense–antisense transcriptional circuitry raises the possibility that the noncoding *cis* polymorphism defining the *FLC* haplotypes might affect *FLC* function through changed *COOLAIR* processing. Here, we show that a single natural noncoding polymorphism can significantly change *COOLAIR* splicing. This SNP is a major contributor to the functional specialization of one of the *FLC* haplotypes. The study has significant implications for understanding the molecular basis of phenotypic evolution.

**Figure 1.**
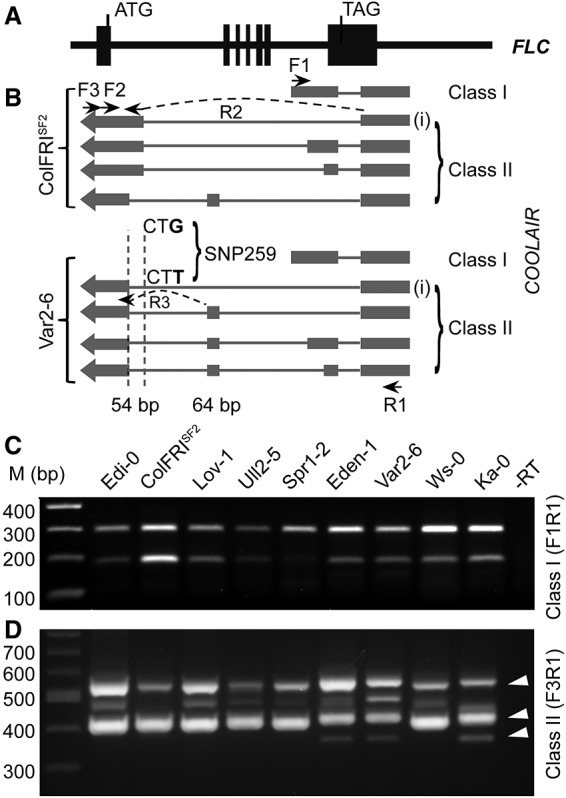
SNP259 in *FLC* influences *COOLAIR* splicing. (*A*,*B*) Schematic illustration of alternative *COOLAIR* splicing and polyadenylation in Col FRI^SF2^ (FRI from SF-2 accession) and Var2-6. (Black boxes) *FLC* exons; (grey boxes) *COOLAIR* exons. The position of SNP259 (Col-0/Var2-6: G/T) is indicated; the nucleotides CT show the splice acceptor site for *COOLAIR* class II transcripts (“i” is marked). Arrows show PCR primers used to assay splicing; arrows plus a dashed line show primers that cover an exon–intron junction. The 54-base-pair (bp) shift of the *COOLAIR* acceptor site is highlighted with two vertical dashed lines, and the 64-bp additional exon is marked. (*C*) The *COOLAIR* class I splicing pattern is the same in a set of accessions representing the different *FLC* haplotypes. The RT–PCR primers F1 and R1 are as shown in *B*. For class I, the lower band is due to mispriming of the F1 oligo and represents unspliced class I *COOLAIR*. (*D*) *COOLAIR* class II is alternatively spliced in a set of accessions representing different *FLC* haplotypes. The RT–PCR primers F3 and R1 are as shown in *B*. The arrowheads indicate the characteristic *COOLAIR* splicing changes in accessions containing the SNP259T polymorphism.

## Results and Discussion

### *A. thaliana* accessions show variable COOLAIR processing

**Figure 2.**
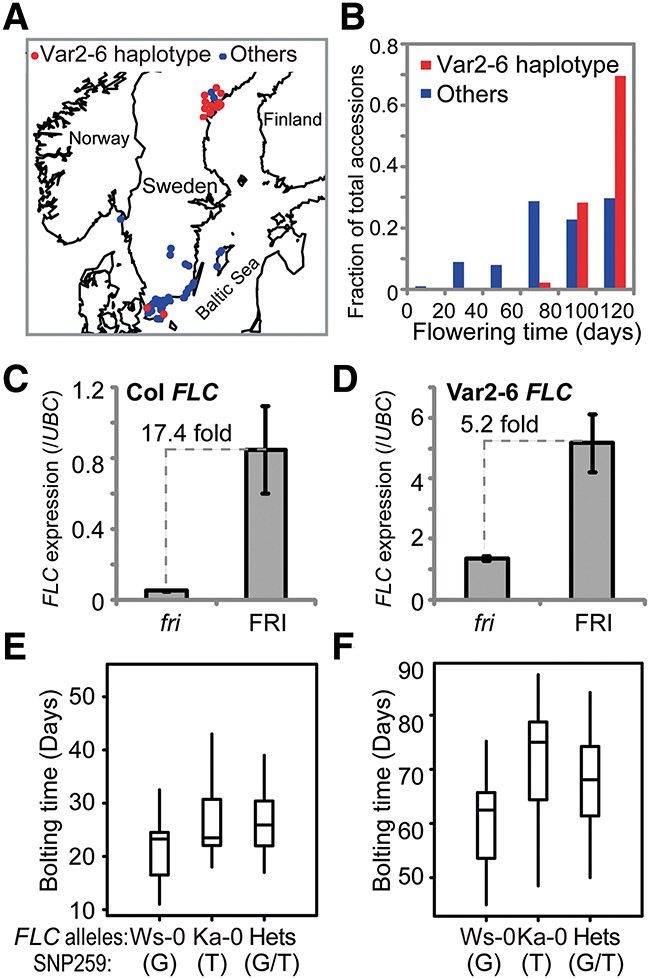
The Var2-6 *FLC* allele confers higher *FLC* expression and later flowering. (*A*) The Var2-6 *FLC* haplotype shows a distinct geographical distribution. Forty-six accessions carrying the Var2-6 haplotype are shown as red dots, and 101 accessions carrying other *FLC* haplotypes are shown as blue dots. The map was generated in R based on the latitude and longitude information of accessions (Supplemental Table S1). (*B*) Accessions carrying the Var2-6 *FLC* haplotype are later flowering than accessions carrying other *FLC* haplotypes. The flowering time of 120 d indicates that the plants did not flower when the experiment was finished. The Kolmogorov-Smirnov (KS) test indicated that the flowering time difference between Var2-6 haplotypes and the other accessions is significant (*P* < 0.001). (*C*) *FLC* expression of the Col allele in plants with and without active FRI. (*D*) *FLC* expression of the Var2-6 allele in plants with and without active FRI. Values in *C* and *D* are means ± SD from three biological repeats. The fold change in *FLC* expression between Col and Var2-6 *FLC* in *fri* is 28, and the fold change in *FRI* is 6. (*E*,*F*) SNP259T is associated with late flowering in an F2 population generated from a cross between Ka-0 and Ws-0 in *fri* (*E*) and *FRI* (*F*) homozygotes. The KS test indicated that the flowering time difference between plants containing the Ka-0 *FLC* allele and the Ws-0 allele is significant (*P* < 0.05).

We surveyed different *FLC* haplotypes for altered splicing patterns of *COOLAIR* (Fig. 1A,B) and identified one haplotype characterized by the accessions Var2-6 and Eden-1 with a distinct *COOLAIR* class II splicing profile but unchanged class I profile (Fig. 1C,D). The accessions containing this haplotype are predominantly found in northern Sweden (Fig. 2A; Supplemental Table S1) and are generally later flowering than other *A. thaliana* accessions (Fig. 2B; Supplemental Table S1), and all consistently show higher than average *FLC* expression (Li et al. 2014). We introgressed one representative allele from this haplotype (from the Var2-6 accession) into Columbia and then compared expression levels in the presence and absence of FRIGIDA (FRI), the major up-regulator of *FLC* expression (Lee et al. 1994; Johanson et al. 2000). The Var2-6 allele was more strongly expressed than the Col allele in a common *fri* background, with the relative up-regulation by FRI higher for the Col (17.4-fold) than the Var-2-6 (5.2-fold) allele (Fig. 2C,D). This genetic interaction suggests that *cis* polymorphism in the Var2-6 *FLC* allele contributes to a higher expression level, potentially through a mechanism similar to FRI function.

### SNP259 affects splicing of COOLAIR

DNA sequence comparison between the Var2-6 and Col-0 *FLC* alleles revealed 43 polymorphisms. One was in the *FLC* coding region and introduces a synonymous nucleotide change in exon 7, so the Var2-6 *FLC* allele encodes a protein identical to Col-0 (Supplemental Table S2). The other polymorphisms were in noncoding regions with one, SNP259 Col-0/Var2-6: G/T, located next to the acceptor splice site of the intron of class II-i *COOLAIR* (Fig. 1B; Supplemental Table S2; Liu et al. 2007, 2010; Marquardt et al. 2014). The functional relevance of SNP259 was explored through analysis of an F2 population from a cross between accessions Ka-0 and Ws-0. These accessions have highly related *FLC* alleles differing only in SNP259 (Ws-0/Ka-0: G/T) and five other polymorphisms within a 22-base-pair (bp) region (Ws-0/Ka-0: C-AAA/TTTTT). The T nucleotide at SNP259 associated with later flowering (Fig. 2E,F) and a *COOLAIR* splicing profile similar to that in Var2-6 (Fig. 1D). These analyses implicated SNP259T in the increased expression of *FLC*, potentially through an influence on *COOLAIR* splicing.

**Figure 3.**
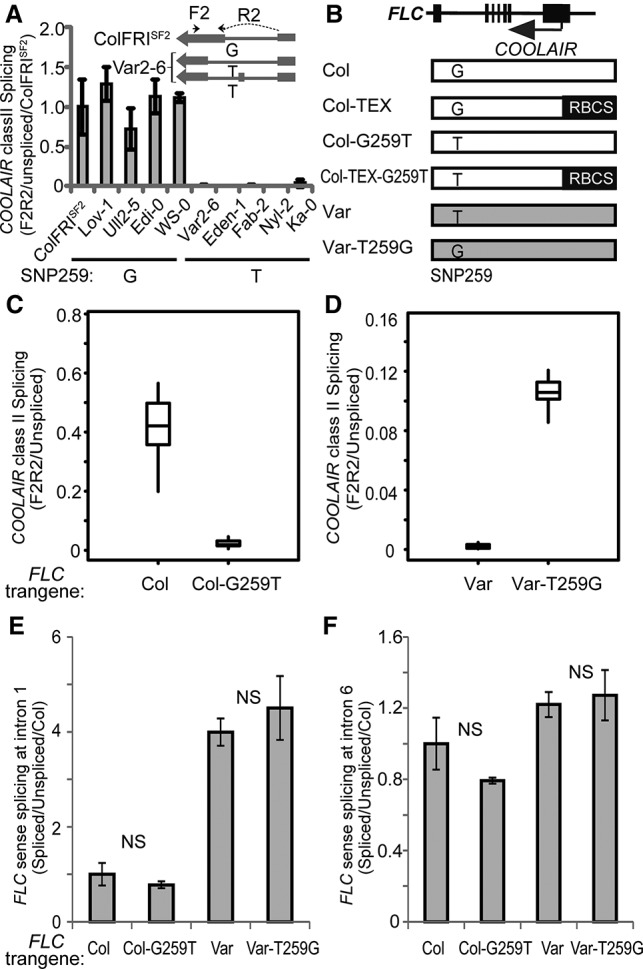
SNP259 specifically regulates *COOLAIR* alternative splicing. (*A*) Accessions with nucleotide SNP259T show reduced *COOLAIR* class II-i splicing compared with those containing SNP259G. Splicing efficiency was measured with quantitative PCR (qPCR) using the primers F2R2 and normalized to unspliced *COOLAIR* transcript levels at the same region. Next, the splicing efficiency was normalized to the value in ColFRI^SF2^. The *inset* shows the position of primer F2R2 for qPCR analysis of *COOLAIR* variants in ColFRI^SF2^ and Var2-6. Multiple accessions containing different *FLC* haplotypes are shown (Li et al. 2014). (*B*) Schematic illustration of the reciprocal SNP259 constructs in Col, Var2-6 *FLC* alleles, and Col *FLC* with RBCS terminator replacement at the *COOLAIR* promoter region. *FLC* and *COOLAIR* are illustrated at the *top*. RBCS in black bars shows the replacement of the *COOLAIR* promoter. The Col-0 and Var2-6 *FLC* alleles are indicated with white and grey boxes. (*C*) G259T mutation in Col *FLC* caused decreased *COOLAIR* class II-i splicing in multiple randomly selected transgenic lines. (*D*) T259G mutation in Var2-6 *FLC* caused increased *COOLAIR* class II-i splicing in multiple randomly selected transgenic lines. In *C* and *D*, each box plot shows the qPCR data of 10 randomly selected independent transgenic plants for each construct. (*E*) G259T heterogeneity did not influence *FLC* sense intron 1 splicing. The Student's *t*-tests indicated that the *FLC* sense intron 1 splicing between Col and Col-G259T or Var and Var-T259G are not significant ([NS]*P* > 0.05). (*F*) G259T heterogeneity did not influence *FLC* sense intron 6 splicing. Values in *A*, *E*, and *F* are means ± SD from three biological repeats. The Student's *t*-tests indicated that the *FLC* sense intron 6 splicing between Col and Col-G259T or between Var and Var-T259G is not significant ([NS] *P* > 0.05).

**Figure 4.**
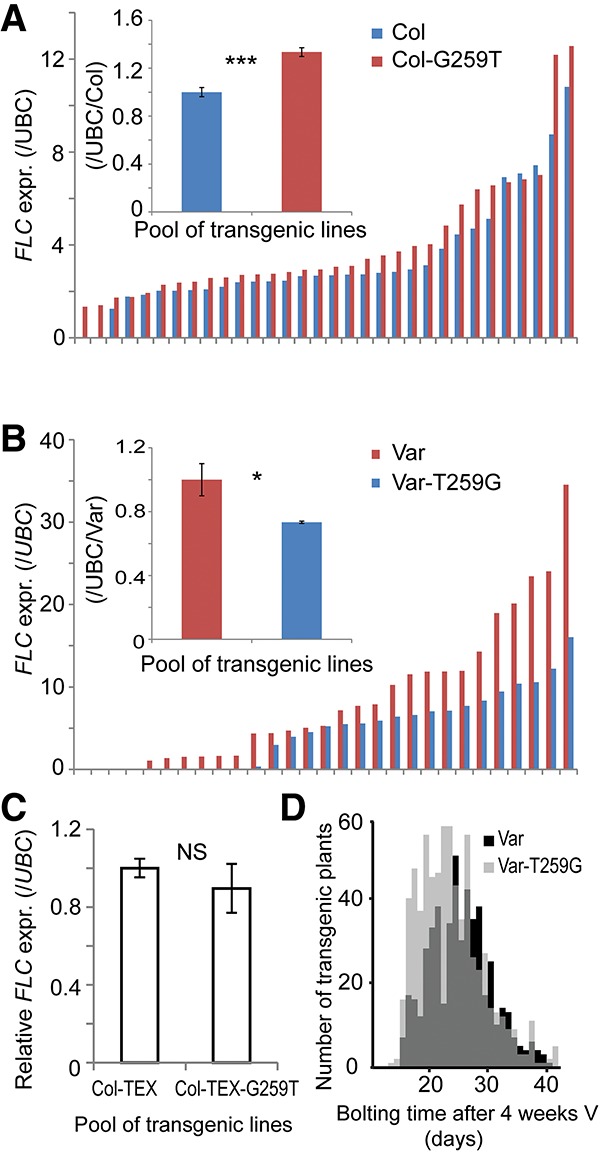
G259T heterogeneity affects *FLC* expression in a *COOLAIR*-dependent mechanism. (*A*) The G259T mutagenesis in Col *FLC* increased *FLC* expression, as assayed using a pool of 48 independent transgenic lines (Student's *t*-test, [***] *P* < 0.001) and 32 randomly selected independent transgenic lines. (*B*) T259G mutagenesis in Var2-6 *FLC* decreased *FLC* expression, as assayed using a pool of 48 independent transgenic lines (Student's *t*-test, [*] *P* < 0.05) and 29 randomly selected independent transgenic lines. (*C*) G259T did not change *FLC* expression in a *COOLAIR* terminator exchange construct, as assayed using pools from 21 and 32 independent transgenic lines: Col-TEX and Col-TEX-G259T (Student's *t*-test, [NS] *P* = 0.216). (*D*) Flowering time comparison of Var/*FRI flc-2* and Var-T259G/*FRI flc-2* of plants given 4 wk of vernalization. Forty-five and 60 independent transgenic lines were scored, with eight individuals of each line included. The KS test indicated that the difference between transgenic lines is significant (*P* < 0.001). In *A*–*C*, values are means ± SD from three biological repeats. All of the transgenic plants shown in *A*–*D* were in a *FRI flc-2* background.

The SNP259T polymorphism was found to significantly reduce use of the splice acceptor site of the class II-i *COOLAIR* intron (Fig. 3A) and increase use of an acceptor site 54 bp downstream (Fig. 1B). This change caused additional alternative splicing of *COOLAIR* and inclusion of an additional 64-bp exon rarely found in *COOLAIR* transcripts of the Col haplotype (Fig. 1B). All of the accessions containing the SNP259T polymorphism showed this additional *COOLAIR* exon (Fig. 3A). To confirm that the G259T polymorphism adjacent to the *COOLAIR* splice acceptor site was the cause of these functional differences, we undertook mutagenesis experiments, reciprocally mutating Col-0 *FLC* to G259T and Var2-6 *FLC* to T259G (Fig. 3B). The Col-G259T mutation changed the pattern of *COOLAIR* splicing so that it contained the additional exon, resembling Var2-6 *FLC* (Fig. 3C; Supplemental Figs. S1, S2A). The Var-T259G changed the pattern of *COOLAIR* splicing so that it resembled Col *FLC* (Fig. 3D; Supplemental Figs. S1, S2B). No difference was detected in *FLC* sense transcript splicing (Fig. 3E,F). The G259T mutation caused an increase, and the T259G mutation caused a decrease in *FLC* expression in transgenic plants (Fig. 4A,B). Taken together, the results indicate that the single G259T polymorphism contributes to increased *FLC* expression through specific modulation of *COOLAIR* class II splicing (Fig. 1B).

To assess whether the G259T polymorphism regulated *FLC* expression directly as well as indirectly via *COOLAIR*, we generated the G-to-T mutation at nucleotide 259 in an allele of *FLC* attenuated in the production of *COOLAIR*. We previously generated a Col *FLC* terminator exchange transgene (Col-TEX) in which the *COOLAIR* promoter had been replaced with an RBCS 3′ terminator region. Production of class I and class II *COOLAIR* transcripts was disrupted in this transgene, although some unspliced nascent antisense transcript was still produced (Supplemental Fig. S3; Csorba et al. 2014; Marquardt et al. 2014; Wang et al. 2014). The G259T polymorphism was introduced into Col-TEX to produce Col-TEX-G259T (Fig. 3B). Plants carrying Col-TEX-G259T expressed *FLC* at the same level as and flowered at a time similar to those carrying the wild-type Col-TEX transgene (Fig. 4C; Supplemental Fig. S4), indicating that the G259T up-regulation of *FLC* expression requires aspects of *COOLAIR* disrupted in Col-TEX. Since the G259T polymorphism increases *FLC* expression, we also asked whether it affected vernalization response. Transgenic lines carrying the Var2-6 *FLC* transgene showed higher *FLC* expression before, during, and after vernalization compared with lines carrying a Var-T259G *FLC* transgene (Supplemental Fig. S5). They also flowered considerably later after 4-wk vernalization (Fig. 4D). The SNP259T polymorphism thus increases *FLC* expression in all conditions and as such would confer a requirement for longer vernalization in natural conditions.

### COOLAIR splicing affects the degree of capping of the FLC nascent transcript

**Figure 5.**
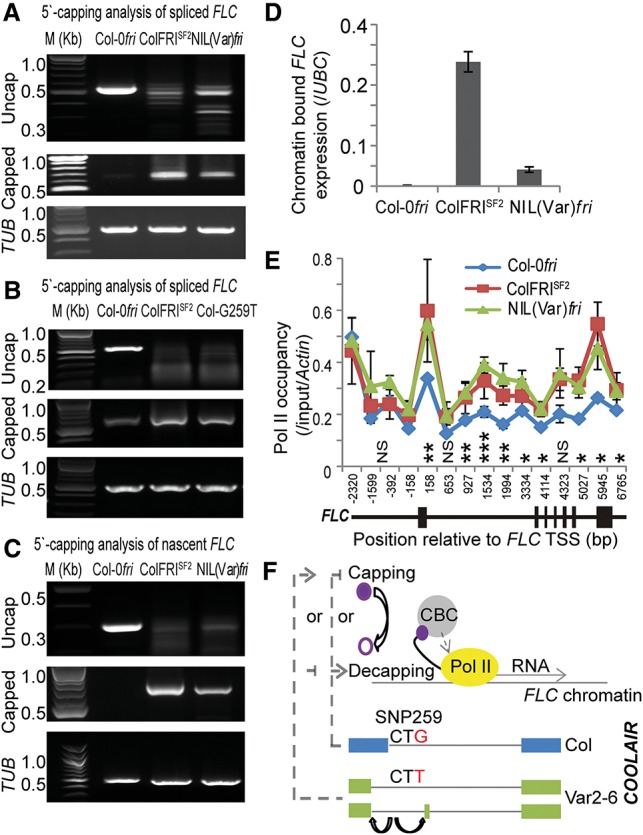
*COOLAIR* alternative splicing links cotranscriptionally to changed transcription and capping of the *FLC* nascent transcript. (*A*) FRI and the Var2-6 *FLC* allele both alter the proportion of transcripts with a 5′ cap. NIL(Var)*fri* is the near isogenic line (NIL) with Var2-6 *FLC* introgressed into a Col-0 *fri* background. (*B*) The single SNP259T polymorphism similarly alters the proportion of transcripts with a 5′ cap. The transgenic Col-G259T plants are in a Col *flc-2 fri* background. The amplified fragment from the uncapped transcript initiates at 5′-AATCAAGCGAATTGAGAACA-3′. (*C*) The 5′ capping analysis of chromatin-bound nascent *FLC*. The amplified fragment from the uncapped transcript initiates at 5′-AAAAAACAATTAATATACCG-3′. In *A*–*C*, *TUBULIN* was used as an internal control. (*D*) Comparison of chromatin-bound nascent *FLC* expression between Col-0*fri*, ColFRI^SF2^, and NIL(Var)*fri*. (*E*) Chromatin immunoprecipitation (ChIP) analysis shows that total RNA polymerase II (Pol II) is more enriched over *FLC* in NIL(Var)*fri* and ColFRI^SF2^ compared with Col-0*fri*. *ACTIN* was used as an internal control. The position of qPCR primers is relative to the *FLC* transcription start site (TSS). In *D* and *E*, values are means ± SD from three biological repeats (Student's *t*-test, [*] *P* < 0.05; [**] *P* < 0.01; [***] *P* < 0.001). (*F*) Model of how SNP259 polymorphisms cotranscriptionally regulate *FLC* expression (Hossain et al. 2013). (Solid purple circle) The 5′ cap at *FLC* transcripts; (open purple circle) an uncapped *FLC* transcript; (CBC) the cap-binding complex; (dashed lines) proposed effects of *COOLAIR* on capping/decapping.

Our earlier genetic analysis suggested that *cis* polymorphism in the Var2-6 *FLC* allele might up-regulate *FLC* expression through a mechanism similar to FRI function (Fig. 2C,D). FRI increases the proportion of *FLC* transcripts carrying a 5′ cap (Geraldo et al. 2009), a structure that influences the cotranscriptional fate of nascent transcripts and the stability and translation of spliced transcripts. We compared the 5′ capping of *FLC* transcripts in different genotypes using an RNA adaptor ligation-mediated PCR assay (Supplemental Fig. S6; Geraldo et al. 2009) and found that a higher proportion of the Var2-6 *FLC* transcripts have a 5′ cap, similar to the functional effect of *FRI* (Fig. 5A). Mutation of SNP259 G to T in the Col *FLC* allele recapitulated the Var2-6 molecular phenotype (Fig. 5B); thus, a single polymorphism (from the 43 that distinguish the Var2-6 from Col *FLC*) is sufficient to change the proportion of *FLC* transcripts carrying a 5′ cap. Changes in *COOLAIR* processing therefore appear to influence *FLC* expression via a mechanism involving the level of capping of the *FLC* transcript.

Capping/decapping reactions occur on transcripts in both the cytoplasm and the nucleus (Ramaiah et al. 2012). Analysis of chromatin-bound *FLC* RNA showed that the SNP259 effect on capping occurs on the *FLC* nascent transcript in the nucleus (Fig. 5C) and is associated with higher levels of chromatin-bound *FLC* transcript (Fig. 5D). We interpret these data as suggesting that SNP259 impacts *FLC* expression through a cotranscriptional mechanism linking antisense splicing with sense strand transcription/capping. Chromatin immunoprecipitation (ChIP) experiments showed that RNA polymerase II (Pol II) levels were approximately twofold higher at the Var2-6 *FLC* allele introgressed into Col as compared with a Col *FLC* allele, similar to the effect of FRI on Col *FLC* (Fig. 5E). The transgenic plants containing the reciprocal SNP259 polymorphisms showed opposing H3K27me3 and H3K36me3 profiles, reinforcing the conclusion that SNP259 influences transcriptional dynamics at *FLC* (Supplemental Fig. S7A,B; Supplemental Table S3). These data are all consistent with the primary effect of SNP259 resulting in a *COOLAIR* variant that up-regulates transcription of *FLC* (or represses less well than other *COOLAIR* class II variants). How a *COOLAIR* variant (an antisense transcript) influences transcription of *FLC* remains to be established, but *COOLAIR* class II variants are associated with chromatin at the 5′ end of *FLC* (Csorba et al. 2014). The SNPG259T may positively influence RNA Pol II dynamics or promote efficient transition to elongation and/or stability of the nascent or spliced transcript, which feeds back to promote transcription (Fig. 5F; Haimovich et al. 2013; Hossain et al. 2013; Bentley 2014).

### A single noncoding polymorphism, SNP259, is a major contributor to the functional specialization of one of the FLC haplotypes

The *FLC* haplotype carrying the SNP259T is predominantly found in accessions that occur in the high latitudes of northern Sweden (Li et al. 2014), where plants experience long cold seasons (Fig. 2A; Supplemental Table S1; climate data from north and south Sweden found at http://www.sweden.climatemps.com). Higher *FLC* expression and an increased vernalization requirement are likely to be adaptively important in these regions, enabling plants to avoid precocious flowering before winter is over. This significant change in reproductive timing behavior is the result of a single polymorphism in a noncoding region of a locus. This should provide an important paradigm in the many studies associating polymorphism with phenotypic variation. It will also influence the debate over the different types of molecular variation that underpin adaptive evolution (Halligan et al. 2013).

## Materials and methods

### Plant materials and growth conditions

Transgenic lines were obtained, and plants grown as described previously (Coustham et al. 2012). Vernalization was performed at 5°C under short day (8 h of light) conditions, and the vernalized plants were moved back to warm growth conditions afterward. Flowering time was assayed by days to flower. If the plants had not flowered after 120 d, then the experiment was stopped, and the flowering time was scored as 120 d.

### FLC expression analysis

*FLC* expression analysis was performed as described (Coustham et al. 2012). Extensive transgenic line variability was found when comparing independent lines carrying the same *FLC* transgene. In order to average this variability and quantify *FLC* expression, we generated a large number of independent transgenic lines for each transgene and pooled seedlings independently three times for the biological replication for RNA extraction and gene expression analyses.

### Association analysis between FLC alleles and flowering time in an F2 population

An F2 population was generated by crossing the accession Ws-0 with Ka-0 and selfing the resulting F1. Two-hundred-eighty-eight F2 individuals were stratified for 3 d at 5°C and grown after no vernalization in long days (16 h light/8 h dark), and bolting time was assayed. The individuals of the F2 population were genotyped with specific PCR markers for Ws-0 and Ka-0 *FLC* alleles (Forward primer, 5′-GTGTTGTGTGTCCAATGTCCATGT-3′; Reverse primer, 5′-AACCAAAATGCCCTAATCTTGAG-3′) and *FRI* alleles (Forward primer, 5′-TACACAAGGATTTTATCATGGGATTAT-3′; Reverse primer, 5′-GTTTCGACAATCTTCGGTAATTCTC-3′). The association between bolting time and Ws-0 and Ka-0 *FLC* alleles was analyzed in the two subpopulations containing Ws-0 FRI and Ka-0 *fri* separately. The box plot and statistical analysis in this study were performed in Gene Stat edition 9 with default parameters (Ripatti et al. 2009).

### Sequence alignment

*FLC* sequences were aligned and exported with BioEdit package version 7.0.9.0 with default parameters (Hall 1999).

### COOLAIR intron splicing analysis

*COOLAIR* class I was amplified with primers F1 (5′-TGGTTGTTATTTGGTGGTGTG-3′) and R1 (5′-GCCGTAGGCTTCTTCACTGT-3′). Class II was amplified with primers F3 (5′-GTATCTCCGGCGACTTGAAC-3′) and R1. PCR fragments were assayed in agarose gel, cloned into pGEM-T Easy Vector (Promega), and sequenced. For the *COOLAIR* class II-i intron splicing assay, cDNA was synthesized with the RT primer *UBC-RP*, F2, and unspliced *COOLAIR* in distal region LP and quantified with the quantitative PCR (qPCR) primers F2 (5′-CTCCTCCGGCGATAAGTA-3′) and R2 (5′-CTCACACGAATAAGAAAAGTAAAA-3′). Unspliced *COOLAIR* in the distal region was amplified with primers LP (5′-CGCAATTTTCATAGCCCTTG-3′) and RP (5′-CTTTGTAATCAAAGGTGGAGAGC-3′). SNP259/T-mediated class II splicing was quantified with primer F2 and R3 (5′-GAAAAAAACCAAACGGTAAGGAAA-3′). The intron splicing was calculated by normalizing spliced to unspliced *COOLAIR*. The PCR position is shown in Figure 1B. The internal control for qRT–PCR is *UBC* with primers 5′-CTGCGACTCAGGGAATCTTCTAA-3′ and 5′-TTGTGCCATTGAATTGAACCC-3′. The sequences of *COOLAIR* splicing variants in Var2-6 were submitted to GenBank (accession no. KP792767-792771).

### FLC sense splicing analysis

We analyzed the intron splicing in *FLC* introns 1 and 6. For intron 1, 2.5 μg of total RNA was treated with Turbo DNase-free kit (Ambion) and reverse-transcribed with primer 5′-CAAGGCTTTAAGATCATCAGCA-3′. Spliced *FLC* expression was assayed with primers 5′-GAGAGAAGCCATGGGAAGAA-3′ and 5′-AGGTTATCGCCGGAGGAG-3′, and unspliced *FLC* expression was assayed with primers 5′-AAAAGTGGAAATTVAGATGTGCT-3′ and 5′-TTGAAAAGGCCACTGGAAAC-3′. For intron 6, 2.5 μg of total RNA was transcribed with primer 5′-GGAGAGTCACCGGAAGATTG-3′. Spliced *FLC* expression was assayed with primers 5′-AAATGCTGAAAGAAGAGAACCAG-3′ and 5′-GGAGAGTCACCGGAAGATTG-3′, and unspliced *FLC* expression was assayed with primers 5′-TGGTTGTTATTTGGTGGTGTG-3′ and 5′-TCTCCATCTCAGCTTCTGCTC-3′. The intron splicing efficiency was calculated by normalizing the spliced *FLC* expression level to the unspliced *FLC* expression level for intron 1 and intron 6, respectively. The internal control for qRT–PCR was *UBC*.

### Capping analysis of FLC RNA

The capping analysis of spliced *FLC* RNA was performed using the FirstChoice RLM-RACE kit (Life Technologies) following the procedure described previously (Geraldo et al. 2009). After the first round of PCR, the PCR product was treated with 0.5 U of exonuclease I (New England Biolabs) and 0.25 U of alkaline phosphatase (shrimp) (Roche) for 30 min at 37°C and 5 min at 95°C and used for nested PCR. For the 5′ capping analysis of nascent *FLC*, the chromatin RNA was prepared as follows: The nuclei were extracted with Honda buffer (0.44 M sucrose, 1.25% Ficoll, 2.5% dextran T40, 20 mM Hepes KoH at pH 7.4, 10 mM MgCl_2_, 0.5% Triton X-100, 5 mM DTT, 1 mM PMSF, 1× EDTA-free Complete protease inhibitor [Roche], 100 μg/mL yeast tRNA, 40 U/mL Superase In RNase inhibitor [Ambion], 5 mM β-mercaptoethanol) and washed with urea/NP40 buffer (20 mM Tris at pH 8.0, 300 mM NaCl, 7.5 mM MgCl_2_, 0.2 mM EDTA, 1 mM DTT, 1 M Urea, 1% Igepal CA-630, 1× EDTA-free Complete protease inhibitor [Roche], 20 U/mL Superase In RNase inhibitor [Ambion]). Next, the chromatin pellet was extracted with the hot phenol method for RNA preparation (Box et al. 2011). 5′ capping analysis was performed using Turbo DNase-treated chromatin RNA following the same procedure. The *FLC*-specific primers for capping analysis were 5′-CCCATAGCAACTCTATAGATCTCCCGTAA-3′ and 5′-CATCGAGCACGCATCAGATCGTATCAAAC-3′. The nested PCR products of all samples were cloned into pGEM-T Easy Vector (Promega) and sequenced with M13 forward primer to confirm the sequence identity.

### ChIP assay

The histone modification and Pol II occupancy assays were performed as described previously (Wang et al. 2014; Yang et al. 2014). The qPCR primers for the ChIP assay are included in Supplemental Table S2. For histone modification assays, anti-Histone H3 (trimethyl K27) antibody (Millipore, 07-449), anti-Histone H3 antibody (Abcam, ab1791), and anti-Histone H3 (trimethyl K36) antibody (Abcam, ab9050) were used. For the total Pol II occupancy assay, anti-RNA polymerase II CTD repeat YSPTSPS antibody [8WG16] (Abcam, ab817) was used for ChIP analysis.

## Supplementary Material

Supplemental Material
