# Supplementary material for: Phenotypic evolution through variation in splicing of the noncoding RNA COOLAIR
Source: Genes Dev. 2015 Apr 1;29(7):696–701. doi: 10.1101/gad.258814.115 (PMC4387712; doi:10.1101/gad.258814.115)
Supplement: Supplemental Material [file supp_29_7_696__index.html]

Phenotypic evolution through variation in splicing of the noncoding RNA COOLAIR — Supplemental Material 

# Phenotypic evolution through variation in splicing of the noncoding RNA *COOLAIR*

## Supplemental Material

**Files in this Data Supplement:**

- Supplemental Figures and Legends.pdf
- Supplemental Table S1.xlsx
- Supplemental Table S2.docx
- Supplemental Table S3.xlsx
