# Supplementary material for: Phenotypic evolution through variation in splicing of the noncoding RNA COOLAIR
Source: Genes Dev. 2015 Apr 1;29(7):696–701. doi: 10.1101/gad.258814.115 (PMC4387712; doi:10.1101/gad.258814.115)

Supplemental Figure Legends:

Supplemental Fig. S1. RT-PCR analysis on *COOLAIR* class II alternative splicing in SNP259 transgenic lines and natural accessions. The position of PCR primers F3 and R1 are indicated in Figure 1B.

Supplemental Fig. S2. Influence of SNP259 polymorphism on *COOLAIR* class II alternative splicing. Comparison of the alternative splicing profiles of *COOLAIR* class II between *FLC* transgenic plants with reciprocal SNP259 mutations in Col-0 (**A**) and Var2-6 (**B**) *FLC* backgrounds. Each boxplot shows the qPCR data of 10 randomly selected independent transgenic plants for each construct. Primers F2 and R3 are indicated in (**Fig. 1B**).

Supplemental Fig. S3. *COOLAIR* is knocked down in Col-TEX transgenic lines. (**A**) Schematic illustration of *FLC* and representative *COOLAIR* variants and the position of primers for proximal and distal *COOLAIR* variants. The quantitative PCR primers for proximal *COOLAIR*, F1: 5`-TGGTTGTTATTTGGTGGTGTG-3` and R5: 5`-CTTCTGCTCCCACATGATGA-3`. Primers for distal *COOLAIR*, F3: 5`-GTATCTCCGGCGACTTGAAC-3` and R6: 5`-GGATGCGTCACAGAGAACAG-3`. (**B**) *COOLAIR* class I variants were knocked down in Col-TEX transgenic lines compared Col lines. (**C**) *COOLAIR* class II variants were knocked down in Col-TEX transgenic lines compared with Col lines.

Supplemental Fig. S4. The flowering time of Col-TEX/*FRI flc-2* and Col-TEX-G259T/*FRI flc-2* did not show significant difference. 27 independent transgenic lines and 20 independent transgenic lines were analysed; in each line, 8 individuals were

assayed. The Kolmogorov-Smirnov test indicated the flowering time difference between Col-TEX/*FRI flc-2* and Col-TEX-G259T/*FRI flc-2* plants is not significant (NS, P=0.426).

Supplemental Fig. S5 Comparison of *FLC* expression after series of vernalization in representative transgenic plants. NV means non-vernalization, T0 means during cold and T10 means 10 days after cold. Values are means +/- S.D from 3 biological repeats.

Supplemental Fig. S6. Illustration of the method for *FLC* RNA 5' capping analysis. Solid black lines: RNA; Red line: RNA adaptor; Red cross: the RNA ligation does not work because of the 5' cap at RNA end.

Supplemental Fig. S7. Histone modification analysis at *FLC* locus in representative transgenic lines Var/*FRI flc-2* and Var-T259G/*FRI flc-2*. (**A**) ChIP analysis of H3K27me3. (**B**) ChIP analysis of H3K36me3. Only the 5' part of the *FLC* locus is analyzed due to background from *FLC* gene remnants left in the *flc-2* fast-neutron-induced mutant. *STM* and *ACTIN* are the internal controls for ChIP experiments. Values are means +/- S.D from 3 biological repeats.

GENESDEV/2015/258814

Li_Supplemental Fig. S1

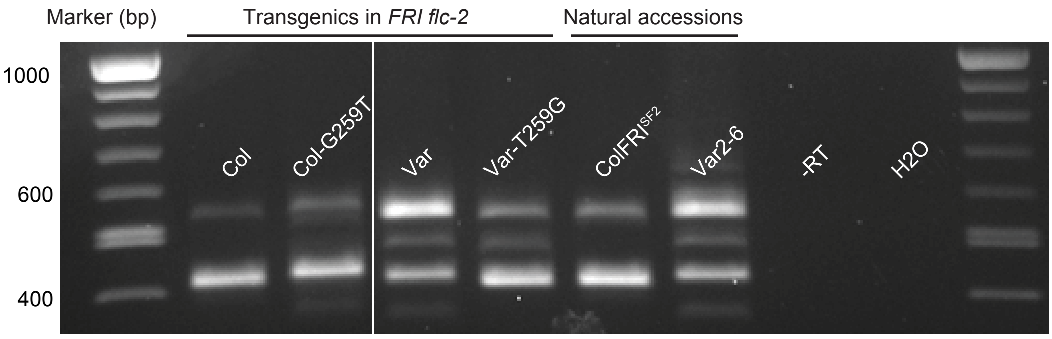

Li_Supplemental Fig. S2

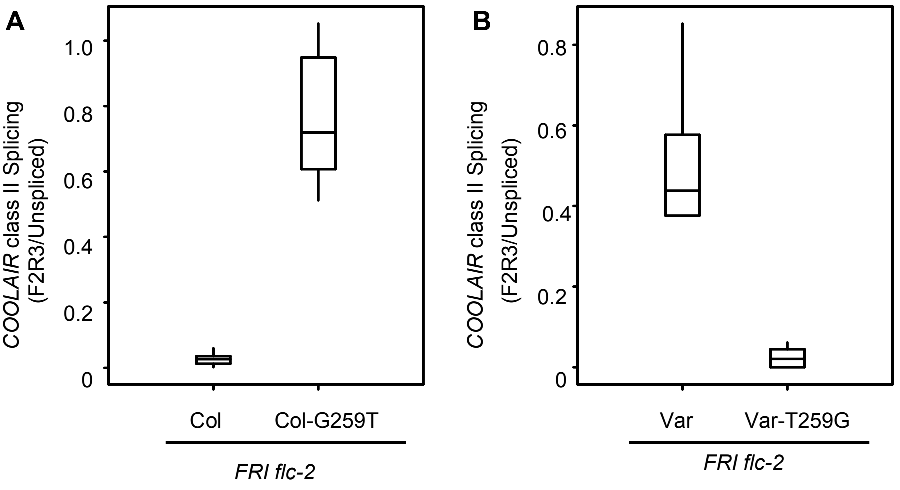

GENESDEV/2015/258814

Li_Supplemental Fig. S3

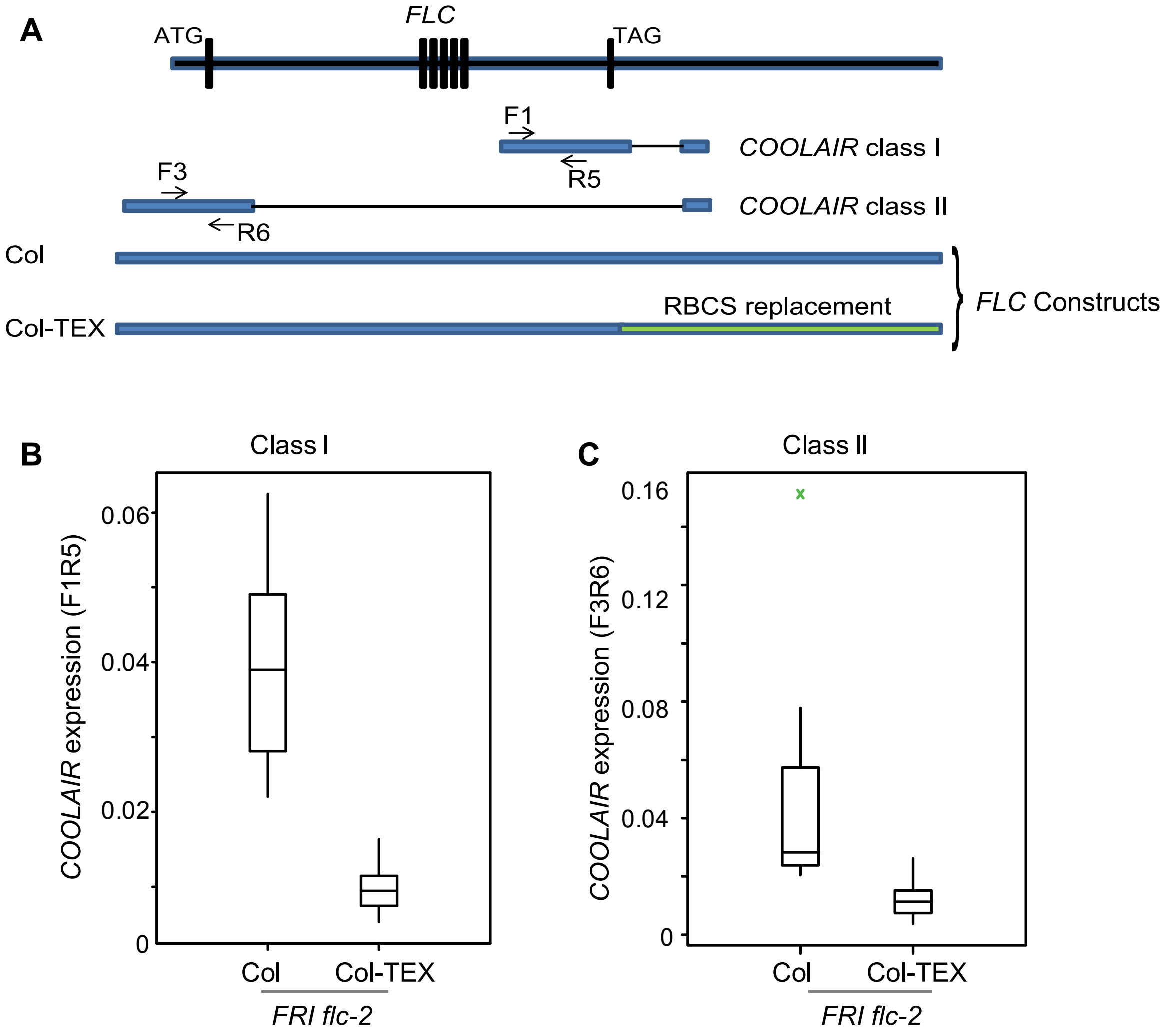

GENESDEV/2015/258814

Li_supplemental Fig. S4

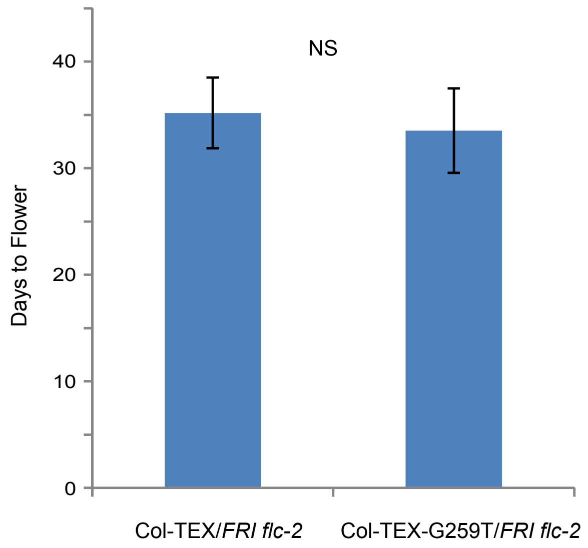

GENESDEV/2015/258814

Li_Supplemental Fig. S5

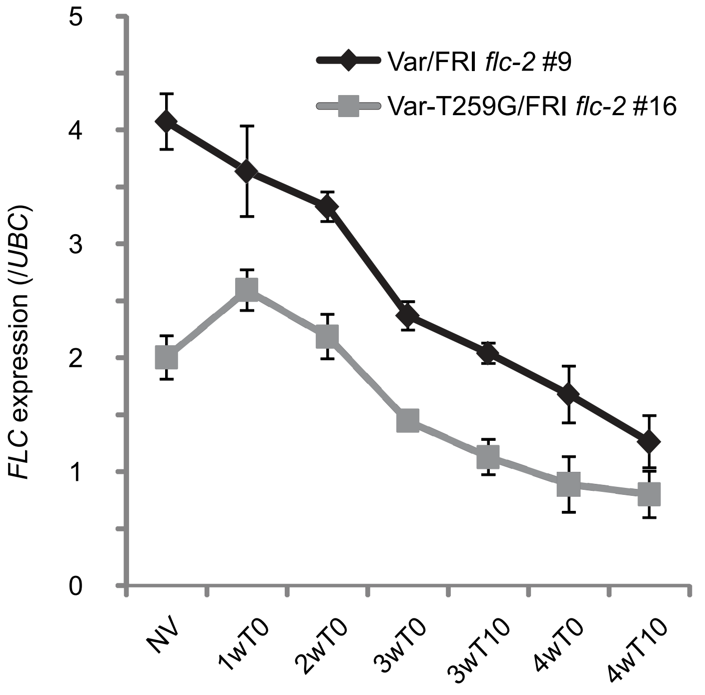

Li_Supplemental Fig. S6

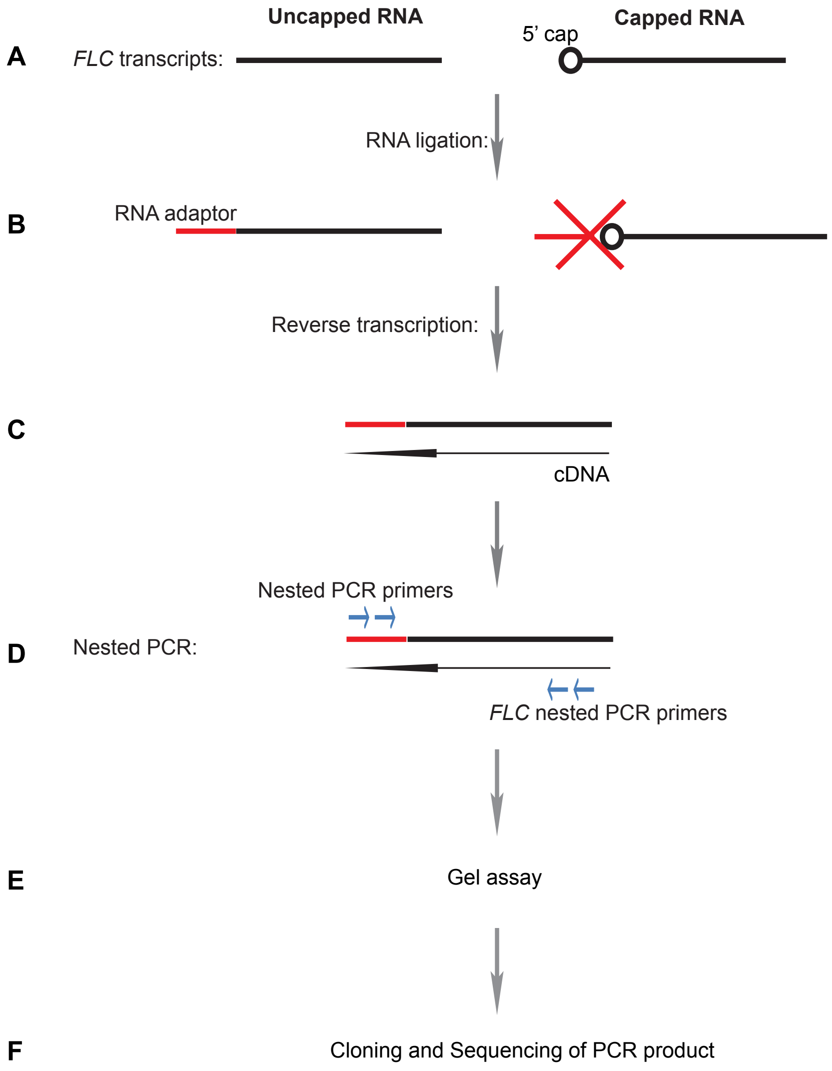

GENESDEV/2015/258814

Li_Supplemental Fig. S7

A

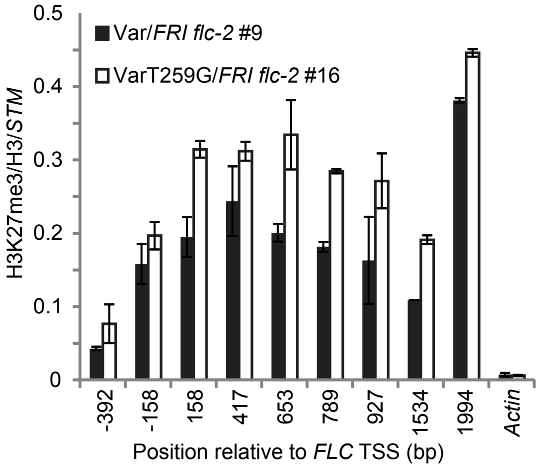

B

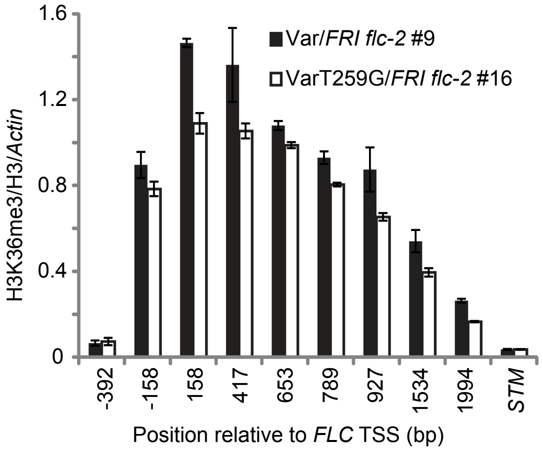

Supplement: Supplemental Material [file supp_gad.258814.115_Supplemental_Figures_and_Legends.pdf]
