# Supplementary material for: Phenotypic evolution through variation in splicing of the noncoding RNA COOLAIR
Source: Genes Dev. 2015 Apr 1;29(7):696–701. doi: 10.1101/gad.258814.115 (PMC4387712; doi:10.1101/gad.258814.115)
Supplement: Supplemental Material [file supp_gad.258814.115_Supplemental_Table_S2.docx]

**Supplemental Table S2 Sequence alignment of Col-0 and Var2-6 *FLC* alleles**

10 20 30 40 50 60 70 80 90 100

....|....|....|....|....|....|....|....|....|....|....|....|....|....|....|....|....|....|....|....|

**Col-0**  **AGCGAGTAAGAAACGAGCAAAGGAATGCAAATTATGTGAATACTATACACCAACAGTGTAGACATGTAGCTACAATGCGGCAATGTTAAAAATAAACTCA**

**Var2-6** **AGCGAGTAAGAAACGAGCAAAGGAATGCAAATTATGTGAATACTATACACCAACAGTGTAGACATGTAGCTACAATGCGGCAATGTTAAAAATAAACTCA**

110 120 130 140 150 160 170 180 190 200

....|....|....|....|....|....|....|....|....|....|....|....|....|....|....|....|....|....|....|....|

**Col-0**  **AATTGGTTTGGAGGGAACAACCTAATGCTTATAAGTACACTTTTGTGGTAAATACAATCCAATTGAAAGTCTTTGTAGGTTTGGTTTGGTCCAATGAAAT**

**Var2-6** **AATTGGTTTGGAGGGAACAACCTAATGCTTATAAGTACACTTTTGTGGTAAATACAATCCAATTGAAAGTCTTTGTAGGTTTGGTTTGGTCCAATGAAAT**

210 220 230 240 250 260 270 280 290 300

....|....|....|....|....|....|....|....|....|....|....|....|....|....|....|....|....|....|....|....|

**Col-0**  **TGTATGCGAGTTGAGTAAAATAACCTTAGTTCAAAACATTAGATATGTAATGGTCTAGATACGATGGTAGCCAAAGATTTGGGTTAAATTTAGAATAAAG**

**Var2-6** **TGTATGCGAGTTGAGTAAAATAACCTTAGTTCAAAACATTAGATATGTAATGGTCTAGATACGATGGTAGCCAAAGATTTGGGTTAAATTTAGAATAAAG**

310 320 330 340 350 360 370 380 390 400

....|....|....|....|....|....|....|....|....|....|....|....|....|....|....|....|....|....|....|....|

**Col-0**  **ATCAAAGAACAAATGACTGACTTCCTTATGTGTGTTTGTTTATGTAAAGTCTTAACTCGTGTCTTGCCAAATTAATAAAAAGGTGCATTATACAATGAAC**

**Var2-6** **ATCAAAGAACAAATGACTGACTTCCTTATGTGTGTTTGTTTATGTAAAGTCTTAACTCGTGTCTTGCCAAATTAATAAAAAGGTGCATTATACAATGAAC**

410 420 430 440 450 460 470 480 490 500

....|....|....|....|....|....|....|....|....|....|....|....|....|....|....|....|....|....|....|....|

**Col-0**  **TAGAGCTTGTTCTCATCAAAAATTTTAACATTAATATAAGTTATTAAAATTTTGTTGATTATATATGATTTCAATATTAAATTATATTTTTTGCTTATAG**

**Var2-6** **TAGAGCTTGTTCTCATCAAAAATTTTAACATTAAAATAAGTTATTAAAATTTTGTTGATTATATATGATTTCAATATTAAATTATATTTTTTGCTTATAG**

510 520 530 540 550 560 570 580 590 600

....|....|....|....|....|....|....|....|....|....|....|....|....|....|....|....|....|....|....|....|

**Col-0**  **CATCAAAACTTCTTGGCACAGCTCCGAGTGTTACTGAAATGTTTGTGTGGCTCCAATAGAAAAGTTAATACCAATCATGAATCATCAATATCGTTACAAA**

**Var2-6** **CATCAAAACTTCTTGGCACAGCTC-GAGTGTTACTGAAATGTTTGTGTGGCTCCAATAGAAAAGTTAATACCAATCATGAATCATCAATATCGTTACAAA**

610 620 630 640 650 660 670 680 690 700

....|....|....|....|....|....|....|....|....|....|....|....|....|....|....|....|....|....|....|....|

**Col-0**  **ATCAGTAATCAGTTTCACCCACTCCGAGTGTTAGTGAAATGTTTGTGTGGCTCCATTAAAAAAGTTAATACCAATCACCAATATCGTTACAAAATTTGTA**

**Var2-6** **ATCAGTAATCAGTTTCACCCACTCCGACTGTTAGTGAAATGTTTGTGTGGCTCCATTAAAAAAGTTAATACCAATCACCAATATCGTTACAAAATTTGTA**

710 720 730 740 750 760 770 780 790 800

....|....|....|....|....|....|....|....|....|....|....|....|....|....|....|....|....|....|....|....|

**Col-0**  **ATCAGTTTCACCCACTTCTTTTCGTCTTGTTACTCGATATAGCCTCACCTTTCAAGTGTCTAATATAACATTTTTTAACAAAATATTACAAAACAAGAAA**

**Var2-6** **ATCAGTTTCACCCACTTCTTTTCGTCTTGTTACTCGATATAGCCTCACCTTTCAAGTGTCTAATATAACATTTTTTAACAAAATATTACAAAACAAGAAA**

810 820 830 840 850 860 870 880 890 900

....|....|....|....|....|....|....|....|....|....|....|....|....|....|....|....|....|....|....|....|

**Col-0**  **AATTATAGTAATTAGATTAATCATATGTAATCTATTACATATAGTAATTTGTATTAGTATCGTTTATTGTGTTACCATTCAAACGGTATAATCTATATAA**

**Var2-6** **AATTATAGTAATTAGATTAATCATATGTAATCTATTACATATAGTAATTTGTATTAGTATCGTTTATTGTGTTACCATTCAAACGGTATAATCTATATAA**

910 920 930 940 950 960 970 980 990 1000

....|....|....|....|....|....|....|....|....|....|....|....|....|....|....|....|....|....|....|....|

**Col-0**  **TTATTAGCAAAACAAAATATAGTTTTAGTAATTAATAAAATTAATATAATGATAGTGATATTCAGAATGTGGTATCTAATCATGTAAAAATATATAATAA**

**Var2-6** **TTATTAGCAAAACAAAATATAGTTTTAGTAATTAATAAAATTAATATAATGATAGTGATATTCAGAATGTGGTATCTAATCATGTAAAAATATATAATAA**

1010 1020 1030 1040 1050 1060 1070 1080 1090 1100

....|....|....|....|....|....|....|....|....|....|....|....|....|....|....|....|....|....|....|....|

**Col-0**  **TAAAATTAGATAAAGAAGAAATTGGTAAAAAATAATTAATGAGATATAAAAGGAAAACAAGCTGATACAAGCATTTCACCAAAAAAAAAACAAGCTGATA**

**Var2-6** **TAAAATTAGATAAAGAAGAAATTGGTAAAAAATAATTAATGAGATATAAAAGGAAAACAAGCTGATACAAGCATTTCACCACAAAAAAA-CAAGCTGATA**

1110 1120 1130 1140 1150 1160 1170 1180 1190 1200

....|....|....|....|....|....|....|....|....|....|....|....|....|....|....|....|....|....|....|....|

**Col-0**  **CAAGCAAAAAAGAATTAGTAACTTTGAGCTATTGCCATATGTGTGGACATTTAAGATTTCTTGTATTTTAGAAATCTTATATATTTCCACAATATATTTA**

**Var2-6** **CAAGCAAAAAAGAATTAGTAACTTTGAGCTATTGCCATATGTGTGGACATTTAAGATTTCTTGTATTTTAGAAATCTTATATATTTCCACAATATATTTA**

1210 1220 1230 1240 1250 1260 1270 1280 1290 1300

....|....|....|....|....|....|....|....|....|....|....|....|....|....|....|....|....|....|....|....|

**Col-0**  **CTACTTTCCTTTATTATTTGTGTTAATCTCCCGAACATTATTATTTCAATACTATCTGAAAAACACATTTTTTTTTATAAAATTTGATGACGTAGGCGAG**

**Var2-6** **CTACTTTCCTTTATTATTTGTGTTAATCTCCCGAACATTATTATTTCAATACTATCTGAAAAACACATTTTTTTTTATAAAATTTGATGACGTAGGCGAG**

1310 1320 1330 1340 1350 1360 1370 1380 1390 1400

....|....|....|....|....|....|....|....|....|....|....|....|....|....|....|....|....|....|....|....|

**Col-0**  **TGGTTCTTTGTTTTTACTATGTAGGCACGACTTTGGTAACACCTACTAATTAACTGCCAAATTTTAAGTTTTGAGAAGTCGGAAGAGTTCAAACCAGTTT**

**Var2-6** **TGGTTCTTTGTTTTTACTATGTAGGCACGACTTTGGTAACACCTACTAATTAACTGCCAAATTTTAAGTTTTGAGAAGTCGGAAGAGTTCAAACCAGTTT**

1410 1420 1430 1440 1450 1460 1470 1480 1490 1500

....|....|....|....|....|....|....|....|....|....|....|....|....|....|....|....|....|....|....|....|

**Col-0**  **TAGGTTTCGATATGTCTACATAGTTCAAAGATGATGTAGAGTGGAGGTTCTTTCTGCAATAGTTCAATCCGTATCGTAGGGGAGGAAAGATAGTTTTCAT**

**Var2-6** **TAGGTTTCGATATGTCTACATAGTTCAAAGATGATGTAGAGTGGAGGTTCTTTCTGCAATAGTTCAATCCGTATCGTAGGGGAGGAAAGATAGTTTTCAT**

1510 1520 1530 1540 1550 1560 1570 1580 1590 1600

....|....|....|....|....|....|....|....|....|....|....|....|....|....|....|....|....|....|....|....|

**Col-0**  **TTAGCAACGAAAGTGAAAACTAAGGCAATGCAAAAGTAGCAAAGACGCTCGTCATGCGGTACACGTGGCAATCTTGTCTTCAAAACACAACGTTTTTATT**

**Var2-6** **TTAGCAAAGAAAGTGAAAACTAAGGCAATGCAAAAGTAGCAAAGACGCTCGTCATGCGGTACACGTGGCAATCTTGTCTTCAAAACGCAACGTTTTTATT**

1610 1620 1630 1640 1650 1660 1670 1680 1690 1700

....|....|....|....|....|....|....|....|....|....|....|....|....|....|....|....|....|....|....|....|

**Col-0**  **CACATATTTGGTTTTTTTGCATCACTCTCGTTTACCCCCAAAAAAAAAAAAATATCTGGCCCGACGAAGAAAAAGTAGATAGGCACAAAAAATAGAAAGA**

**Var2-6** **CACATATTTGGTTTTTTTGCATCACTCTCGTTTACCCC-AAAAAAAAAAAAATATCTGGCCCGACGAAGAAAAAGTAGATAGGCCCAAAAAATAGAAAGA**

1710 1720 1730 1740 1750 1760 1770 1780 1790 1800

....|....|....|....|....|....|....|....|....|....|....|....|....|....|....|....|....|....|....|....|

**Col-0**  **AATAAAGCGAGAAAAGGAAAAAAAAAAATAGAAAGAGAAAACGCTTAGTATCTCCGGCGACTTGAACCCAAACCTGAGGATCAAATTAGGGCACAAAGCC**

**Var2-6** **AATAAAGCGAGAAAAGGAAAAAAAAAAATAGAAAGAGAAAACGCTTAGTATCTCCGGCGACTTGAACCCAAACCTGAGGATCAAATTAGGGCACAAAGCC**

1810 1820 1830 1840 1850 1860 1870 1880 1890 1900

....|....|....|....|....|....|....|....|....|....|....|....|....|....|....|....|....|....|....|....|

**Col-0**  **CTCTCGGAGAGAAGCCATGGGAAGAAAAAAACTAGAAATCAAGCGAATTGAGAACAAAAGTAGCCGACAAGTCACCTTCTCCAAACGTCGCAACGGTCTC**

**Var2-6** **CTCTCGGAGAGAAGCCATGGGAAGAAAAAAACTAGAAATCAAGCGAATTGAGAACAAAAGTAGCCGACAAGTCACCTTCTCCAAACGTCGCAACGGTCTC**

1910 1920 1930 1940 1950 1960 1970 1980 1990 2000

....|....|....|....|....|....|....|....|....|....|....|....|....|....|....|....|....|....|....|....|

**Col-0**  **ATCGAGAAAGCTCGTCAGCTTTCTGTTCTCTGTGACGCATCCGTCGCTCTTCTCGTCGTCTCCGCCTCCGGCAAGCTCTACAGCTTCTCCTCCGGCGATA**

**Var2-6** **ATCGAGAAAGCTCGTCAGCTTTCTGTTCTCTGTGACGCATCCGTCGCTCTTCTCGTCGTCTCCGCCTCCGGCAAGCTCTACAGCTTCTCCTCCGGCGATA**

2010 2020 2030 2040 2050 2060 2070 2080 2090 2100

....|....|....|....|....|....|....|....|....|....|....|....|....|....|....|....|....|....|....|....|

**Col-0**  **AGTACGCCTTTTCCTTACCTGGGTTTTCATTTGTTCCCCCTTTTATCTTCTGTTTTGTGCTCTTTTACTTTTCTGAGAAAATAAAAATAAAAAAACAATT**

**Var2-6** **AGTACGCCTTTTCCTTACCTGGGTTTTCATTTATTCCCCCTTTTATCTTCTGTTTTGTGCTCTTTTACTTTTCTTAGAAAATAAAAATAAAAAAACAATT**

*COOLAIR* class II large intron splicing acceptor site

***SNP259***

2110 2120 2130 2140 2150 2160 2170 2180 2190 2200

....|....|....|....|....|....|....|....|....|....|....|....|....|....|....|....|....|....|....|....|

**Col-0**  **AATATACCGTTTGGTTTTTTTCCGGCGGATCTCTTGTTGTTTCTCGGTTCTGTGTTTGTTTGTGTTTTTTT-CTGCGACCATGATAGATACATGAGATAA**

**Var2-6** **AATATACCGTTTGGTTTTTTTCCGGCGGATCTCTTGTTGTTTCTCGGTTCTGTGTTTGTTTGTGTTTTTTTTCTGCGACCATGATAGATACATGAGATAA**

2210 2220 2230 2240 2250 2260 2270 2280 2290 2300

....|....|....|....|....|....|....|....|....|....|....|....|....|....|....|....|....|....|....|....|

**Col-0**  **CCAAATTTAAGGAAGAACAATGTCGTGAAGAAGCTTTTTAGCTTCTTACTTTTGTTCATTTCTCTCTCTATTTCTTAAAAAAAAAAATTCTGCATGGATT**

**Var2-6** **CCAAATTTAAGGAAGAACAATGTCGTGAAGAAGCTTTTTAGCTTCTTACTTTTGTTCATTTCTCTCTCTATTTCTTAAAAAAAAAAATTCTGCATGGATT**

2310 2320 2330 2340 2350 2360 2370 2380 2390 2400

....|....|....|....|....|....|....|....|....|....|....|....|....|....|....|....|....|....|....|....|

**Col-0**  **TCATTATTTCCTTGGAAAAAAATTGCATGTCATTCACGATTTGTTTGATACGATCTGATGCGTGCTCGATGTTGTTGAGTGAAGTTTCAAGCCATCTTTG**

**Var2-6** **TCATTATTTCCTTGGAAAAAAATTGCATGTCCTTCACGATTTGTTTGATACGATCTGATGCGTGCTCCATGTTGTTGAGTGAAGTTTCAAGCCATCTTTG**

2410 2420 2430 2440 2450 2460 2470 2480 2490 2500

....|....|....|....|....|....|....|....|....|....|....|....|....|....|....|....|....|....|....|....|

**Col-0**  **ATTGTTTCTTACCTTTAGAGATTCCTTAAGTTTTTGAAGAGTTAATTATATATCACAAGACTAATGATTAATGTCTCTTTTCAGAGTGATTAAAATTCAT**

**Var2-6** **ATTGTTTCTTACCTTTAGAGATTCCTTAAGTTTTTGAAGAGTTAATTATATATCACAAGACTAATGATTAATGTCTCTTTTCAGAGTGATTAAAATTCAT**

2510 2520 2530 2540 2550 2560 2570 2580 2590 2600

....|....|....|....|....|....|....|....|....|....|....|....|....|....|....|....|....|....|....|....|

**Col-0**  **TGGATCTCTCGGATTTGTATGCAATGCACTTACGGGAGATCTATAGAGTTGCTATGGGGTTAATGCTGAACAATGTATATATACCACATTGTGCAGCTAT**

**Var2-6** **TGGATCTCTCGGATTTGTATGCAATGCACTTACGGGAGATCTATAGAGTTGCTATGGGGTTAATGCTGAACAATGTATATATACCACATTGTGCAGCTAT**

2610 2620 2630 2640 2650 2660 2670 2680 2690 2700

....|....|....|....|....|....|....|....|....|....|....|....|....|....|....|....|....|....|....|....|

**Col-0**  **TGACTATATAAGACTATTGTTAATCTTCTATGAATTCCTATCTTTGCTGTGGACCTATTACTTGGTGATTATCCAAATTAGTGTTTTTAATTGATTCATA**

**Var2-6** **TGACTATATAAGACTATTGTTAATCTTCTATGAATTCCTATCTTTGCTGTGGACCTATTACTTGGTGATTATCCAAATTAGTGTTTTTAATTGATTCATA**

2710 2720 2730 2740 2750 2760 2770 2780 2790 2800

....|....|....|....|....|....|....|....|....|....|....|....|....|....|....|....|....|....|....|....|

**Col-0**  **TTTTTCATACACAGTAGTTTTGAATTTTGGTAGCTTCAAAAAACTCAGCCTCACAATTAGTACTTACCGCACATATGCTACTTCAGTAACATAATCTGGT**

**Var2-6** **TTTTTCATACACAGTAGTTTTGAATTTTGGTAGCTTCAAAAAACTCAGCCTCACAATTAGTACTTACCGCACATATGCTACTTCAGTAACATAATCTGGT**

2810 2820 2830 2840 2850 2860 2870 2880 2890 2900

....|....|....|....|....|....|....|....|....|....|....|....|....|....|....|....|....|....|....|....|

**Col-0**  **TATCGATTGCGATTCTTTGAATCACAATCGTCGTGTGCTATATATATAACACCTTTTGCTGTACATAAACTGGTCTAATTTTAGACTAATTAAATTTCAT**

**Var2-6** **TATGGATTGCGATTCTTTGAATCACAATCGTCGTGTGCTATATATATAACACCTTTTGCTGTACATAAACTGGTCTAATTTTAGACTAATTAAATTTCAT**

2910 2920 2930 2940 2950 2960 2970 2980 2990 3000

....|....|....|....|....|....|....|....|....|....|....|....|....|....|....|....|....|....|....|....|

**Col-0**  **TGTTCTCTTGGATTTGTATATGCACGTCCGGGAGATTTATAAATAAAATTAGTATGAGGTTAATGGTAAAAAGGATCAAGAAGTTTGGTTTTAAATGTAA**

**Var2-6** **TGTTCTCTTGGATTTGTATATGCACGTCCGGGAGATTTATAAATAAAATTAGTATGAGGTTAATGGTAAAAAGGATCAAGAAGTTTGGTTTTAAATGTAA**

3010 3020 3030 3040 3050 3060 3070 3080 3090 3100

....|....|....|....|....|....|....|....|....|....|....|....|....|....|....|....|....|....|....|....|

**Col-0**  **GCCACATTAATTGGGAAACTATGACTAAAAGATGAATTGGTAATATATACATAATATTTTTAACGAATTTCTCTCCTTTTTATGGGATATGCTATTTAAA**

**Var2-6** **GCCACATTAATTGGGAAACTATGACTAAAAGATGAATTGGTAATATATACATAATATTTTTAACGAATTTCTCTCCTTTTTATGGGATATGCTATTTGAA**

3110 3120 3130 3140 3150 3160 3170 3180 3190 3200

....|....|....|....|....|....|....|....|....|....|....|....|....|....|....|....|....|....|....|....|

**Col-0**  **GATTGTCCAAAGGTTTATAGTTTCCCACTCTTGCAGTTACACACATAGATTTGCCTCATATTTATGTGATTGTATATCAATTATCGCCCTTAATCTTATC**

**Var2-6** **GATTGTCCAAAGGTTTATAGTTTCCCACTCTTGCAGTTACACACATAGATTTGCCTCATATTTATGTGATTGTATATCAATTATCGCCCTTAATCTTATC**

3210 3220 3230 3240 3250 3260 3270 3280 3290 3300

....|....|....|....|....|....|....|....|....|....|....|....|....|....|....|....|....|....|....|....|

**Col-0**  **ATCGTTGTGTTCATTTATGACTTTGTTCCTATTCGTTAAAATTGACAATCCACAACCTCAATCTTTTGTTGTGAAAATCGACAATCACACAACCTTTGTA**

**Var2-6** **ATCGTTGTGTTCATTTATGACTTTGTTCCTATTCGTTAAAATTGACAATCCACAACCTCAATCTTTTGTTGTGAAAATCGACAATCACACAACCTTTGTA**

3310 3320 3330 3340 3350 3360 3370 3380 3390 3400

....|....|....|....|....|....|....|....|....|....|....|....|....|....|....|....|....|....|....|....|

**Col-0**  **TCTTGTGTCTTTTGTCACACAACCTTTGTATCTTGTGTCTTTTGTCATGGAAATTGTCATTCACACAGCCTTGTTTCTTTGGTGCCTCTAGGAAATTGAA**

**Var2-6** **TCTTGTGTCTTTTG------------------------------TCATGGAAATTGTCATTCACACAGCCTTGTTTCTTTGGTGCCTCTAGGAAATTGAA**

3410 3420 3430 3440 3450 3460 3470 3480 3490 3500

....|....|....|....|....|....|....|....|....|....|....|....|....|....|....|....|....|....|....|....|

**Col-0**  **AATCCCACAACACTTGTCTTCATGTAAGAAATACCAACCTCTTTGGTACGGATCTATAATGAATCAATATAATCCTATATATAAGTTGTCAAAATTGAAT**

**Var2-6** **AATCCCACAACACTTGTCTTCATGTAAGAAATACCAACCTCTTTGGTACGGATCTATAATGAATCAATATAATCCTATATATAAGTTGTCAAAATTGAAT**

3510 3520 3530 3540 3550 3560 3570 3580 3590 3600

....|....|....|....|....|....|....|....|....|....|....|....|....|....|....|....|....|....|....|....|

**Col-0**  **CTGGTGTAGTGTCTACTACAACCCTCCAATATAATAACCAAATGGTTGTAGTAGTTTGGCCATGTTGGTCAAGATCGCTGGCCGATTCTCACTTGATGCA**

**Var2-6** **CTGGTGTAGTGTCTACTACAACCCTCCAATATAATAACCAAATGGTTGTAGTAGTTTGGCCATATTGGTCAAGATCGCTGGCCGATTCTCACTTGATGCA**

3610 3620 3630 3640 3650 3660 3670 3680 3690 3700

....|....|....|....|....|....|....|....|....|....|....|....|....|....|....|....|....|....|....|....|

**Col-0**  **TACTTTGTTAGGATTTGTTCACCCCTAGTTAGGTCCAGCCTTGGAATTGTCGAGACACCTGACTAGAACTCCTGGTCTTAATTATGATTTAATAAAGAAG**

**Var2-6** **TACTTTGTTAGGATTTGTTCACCCCTAGTTAGGTCCAGCCTTGGAATTGTCGAGACACCTGACTAGAACTCCTGGTCTTAATTATGATTTAATAAAGAAG**

3710 3720 3730 3740 3750 3760 3770 3780 3790 3800

....|....|....|....|....|....|....|....|....|....|....|....|....|....|....|....|....|....|....|....|

**Col-0**  **AAGCCTTTTAGAACGTGGAACCCTTAGTTACTCAGTTACTCTTTTTGCATACTTAGGTTGATGCAAAGAGCTTAACTTCACAATAGGACTGATATCTATT**

**Var2-6** **AAGCCTTTTAGAACGTGGAACCCTTAGTTACTCAGTTACTCTTTTTGCATACTTAGGTTGATGCAAAGAGCTTAACTTCACAATAGGACTGATATCTATT**

3810 3820 3830 3840 3850 3860 3870 3880 3890 3900

....|....|....|....|....|....|....|....|....|....|....|....|....|....|....|....|....|....|....|....|

**Col-0**  **AACAAAACAAATTAAGTGAAGTTTTGTCAAAATTGTTGGATCTTCTAGGTCAATATGTAGTTTAGTTTTTATCTGTCTTAGTCGCTTCCTTCTATGGAAG**

**Var2-6** **AACAAAACAAATTAAGTGGAGTTTTGTCAAAATTGTTGGATCTTCTAGGTCAATATGTAGTTTAGTTGTTATCTGTCTTAGTCGCTTCCTTCTATGGAAG**

3910 3920 3930 3940 3950 3960 3970 3980 3990 4000

....|....|....|....|....|....|....|....|....|....|....|....|....|....|....|....|....|....|....|....|

**Col-0**  **ATATATTTATAGATATGTGTGATAAGTTTCCTACTAATATTAGTTAGTGTAACTTCAAGGGCAGAAAACTCTTTACTTTTATTGCTTGATTAATTTGGGG**

**Var2-6** **ATATATTTATAGATATGTGTGATAAGTTTCCTACTAATATTAGTTAGTGTAACTTCAAGGGCAGAAAACTCTTTACTTTTATTGCTTGATTAATTTGGGG**

4010 4020 4030 4040 4050 4060 4070 4080 4090 4100

....|....|....|....|....|....|....|....|....|....|....|....|....|....|....|....|....|....|....|....|

**Col-0**  **TTTAAATATAGGAAATTGGAACCTCACAGTTTCTATAAACGAGTAAGTAATTGAATGTGAAATAACAAAATGGAAGACCGGCTTCCTATTCTTAGGAGTC**

**Var2-6** **TTTAAATATAGGAAATTGGAACCTCACAGTTTCTATAAACGAGTAAGTAATTGAATGTGAAATAACAAAATGGAAGACCGGCTTCCTATTCTTAGGAGTC**

4110 4120 4130 4140 4150 4160 4170 4180 4190 4200

....|....|....|....|....|....|....|....|....|....|....|....|....|....|....|....|....|....|....|....|

**Col-0**  **TTTTGATATTTGCAAAAAAAACATATACAATAGAAATATGAGTTTTGTCTAAGACTCGGTCCATGTATTTGGAGTTTGGCTTCCTCATACTTATGGTTAT**

**Var2-6** **TTTTGATATTTGCAAAAAAAACATATACAATAGAAATATGAGTTTTGTCTAAGACTCGGTCCATGTATTTGGAGTTTGGCTTCCTCATACTTATGGTTAT**

4210 4220 4230 4240 4250 4260 4270 4280 4290 4300

....|....|....|....|....|....|....|....|....|....|....|....|....|....|....|....|....|....|....|....|

**Col-0**  **CTGGTTACCGCCACATCATCATTATCATCTTATGGGTCATCAATACTAGCTCTATCGCTGGAAAAAACCTTGTCCTCAAGGTTCATTGAAAAATCCGAAA**

**Var2-6** **CTGGTTACCGCCACATCATCATTATCATCTTATGGGTCATCAATACTAGCTCTATCGCTGGAAAGAACCTTGTCCTCAAAGTTCATTGAAAAATCCGAAA**

4310 4320 4330 4340 4350 4360 4370 4380 4390 4400

....|....|....|....|....|....|....|....|....|....|....|....|....|....|....|....|....|....|....|....|

**Col-0**  **AGTTTTCTCGTATATGTTGATATGGTATTACTTACAAACAAAGAGCTGATGTTACCAATTTTGACACGAGATTACTAATGAACTCATGAAAGAGGCGTTT**

**Var2-6** **AGTTTTCTCGTATATGTTGATATGGTATTACTTACAAACAAAGAGCTGATGTTACCAATTTTGACACGAGATTACTAATGAACTCATGAAAGAGGCGTTT**

4410 4420 4430 4440 4450 4460 4470 4480 4490 4500

....|....|....|....|....|....|....|....|....|....|....|....|....|....|....|....|....|....|....|....|

**Col-0**  **TTAAAAAATTCTTTTTAAAACTGGGATACAAAAAGAAAAGAGGTAACTAATAATTTGATACCATTGTTCGTAGTCCTGATCAAATGTTATAAGGGTAAAC**

**Var2-6** **TAAAAAAATTCTTTTTAAAACTGGGATACAAAAAGAAAAGAGGTAACTAATAATTTGATACCATTGTTCGTAGTCCTGATCAAATGTTATAAGGGTAAAC**

4510 4520 4530 4540 4550 4560 4570 4580 4590 4600

....|....|....|....|....|....|....|....|....|....|....|....|....|....|....|....|....|....|....|....|

**Col-0**  **ATGATAGAAAATAGAGGGTAAATAGGTTTTGTTCTTATAATGGTTTTGATAACACGCTTTGTAAAGGATATAGGTGTTTTTTGATGCTAAAAGTTGTGGT**

**Var2-6** **ATGATAGAAAATAGAGGGTAAATAGGTTTTGTTCTTATAATGGTTTTGATAACACGCTTTGTAAAGGATATAGGTGTTTTTTTATGCTAAAAGTTGTGGT**

4610 4620 4630 4640 4650 4660 4670 4680 4690 4700

....|....|....|....|....|....|....|....|....|....|....|....|....|....|....|....|....|....|....|....|

**Col-0**  **ATGGATCAAAACCAAAATGGAAGCTCTGAATCTCTGATAGAGGTTGCAATTAGAATTATATAAGTTAATTTGCAAATGAATTGGAAGCAGTCTTCCACTA**

**Var2-6** **ATGGATCCAAACCAAAATGGAAGCTCTGAATCTCTGATAGAGGTTGCAATTAGAATTATATAAGTTAATTTGCAAATGAATTGGAAACAGTCTTCCACTA**

4710 4720 4730 4740 4750 4760 4770 4780 4790 4800

....|....|....|....|....|....|....|....|....|....|....|....|....|....|....|....|....|....|....|....|

**Col-0**  **TTTGCTATTGTTAGGGAAGTCTTTCAGTTAATTTCAGAAAATTAAGAGAAATATGACTTTCTAGACTCAGTCTGTGTACTTGGAATTTTACTTCGGTTTA**

**Var2-6** **TTTGCTATTGTTAGGGAAGTCTTTCAGTTAATTTCAGAAAATTAAGAGAAATATGACTTTCTAAACTCAGTCTGTGTACTTGGAATTTTACTTCGGTTTA**

4810 4820 4830 4840 4850 4860 4870 4880 4890 4900

....|....|....|....|....|....|....|....|....|....|....|....|....|....|....|....|....|....|....|....|

**Col-0**  **CTTCCATGTCATCACATTGTGGCTCATCAATATATGTGTGTATATACATTCATGAGTATATATGATTTCTGGAAAAATAAAAATTGCTTGTTTGCATTTA**

**Var2-6** **CTTCCATGTCATCACATTGTGGCTCATCAATATATGTGTGTATATACATTCATGAGTATATATGATTTCTGGAAAAATAAAAATTGCTTGTTTGCATTTA**

4910 4920 4930 4940 4950 4960 4970 4980 4990 5000

....|....|....|....|....|....|....|....|....|....|....|....|....|....|....|....|....|....|....|....|

**Col-0**  **AGATTGGGGCTGCGTTTACATTTTATATTGCATCAATTATTTCAACATAGATTCACAAACATAAATGCATAGAAACAATCTGGACAGTAGAGGCTTATGT**

**Var2-6** **AGATTGGGGCTGCGTTTACATTTTATATTGCATCAATTATTTCAACATAGATTCACAAACATAAATGCATAGAAACAATCTGGACAGTAGAGGCTTATGT**

5010 5020 5030 5040 5050 5060 5070 5080 5090 5100

....|....|....|....|....|....|....|....|....|....|....|....|....|....|....|....|....|....|....|....|

**Col-0**  **TTAGGGTTCTTATGTACCTTAACTAGTTTGACTTTAAGTTAATCAAAGCCAGCGCTATCACTAAACTTTATCTGTATGCCTTTGTATGACTTTTCTTTGA**

**Var2-6** **TTAGGGTTCTTATGTACCTTAACTAGTTTGACTTTAAGTTAATCAAAGCCAGCGCTATCACTAAACTTTATCTGTATGCCTTTGTATGACTTTTCTTTGA**

5110 5120 5130 5140 5150 5160 5170 5180 5190 5200

....|....|....|....|....|....|....|....|....|....|....|....|....|....|....|....|....|....|....|....|

**Col-0**  **GGGAAAATGTCATTTTCAATCTGCCGAAATATATAATAAATACATGTTAGCCCACATAATTCATTGGATAACTAATCTTTGAGCAATTTTTGGTAAATGT**

**Var2-6** **GGGAAAATGTCATTTTCAATCTGCCGAAATATATAATAAATACATGTTAGCCCACATAATTCATTGGATAACTAATCTTTGAGCAATTTTTGGTAAATGT**

5210 5220 5230 5240 5250 5260 5270 5280 5290 5300

....|....|....|....|....|....|....|....|....|....|....|....|....|....|....|....|....|....|....|....|

**Col-0**  **TTTGGTTCTTTTCTTTTCTTGAGAGAGAAAAAAAATATCAGATATTATTAAATATTGCTTACAAAGCTAAGAACAAGTTAAAACTTTTTTGAAAAAGTGG**

**Var2-6** **TTTGGTTCTTTTCTTTTCTTGAGAGAGAAAAAAAATATCAGATATTATTAAATATTGCTTACAAAGCTAAGAACAAGTTAAAACTTTTTTGAAAAAGTGG**

5310 5320 5330 5340 5350 5360 5370 5380 5390 5400

....|....|....|....|....|....|....|....|....|....|....|....|....|....|....|....|....|....|....|....|

**Col-0**  **AAATTCAGATGTGCTACTGCTTAAACATGAATATTAAGATTATTGTTTTTCTGAAATGTTACGAATACTAGCGTGTTATATATATGTAAAAGGTAAGGTG**

**Var2-6** **AAATTCAGATGTGCTACTGCTTAAACATGAATATTAAGATTATTGTTTTTCTGAAATGTTACGAATACTAGCGTGTTATATATATGTAAAAGGTAAGGTG**

5410 5420 5430 5440 5450 5460 5470 5480 5490 5500

....|....|....|....|....|....|....|....|....|....|....|....|....|....|....|....|....|....|....|....|

**Col-0**  **TTCTCTCAATGTTTCATAGTTTCCAGTGGCCTTTTCAAGGGTTAGCTAGTAGTTTTGATCCTAACATATTTTTATTTTTTTTGTCATCTCTCCAGCCTGG**

**Var2-6** **TTCTCTCAATGTTTCATAGTTTCCAGTGGCCTTTTCAAGGGTTAGCTAGTAGTTTTGATCCTAACATATTTTTATTTTTTTTGTCATCTCTCCAGCCTGG**

5510 5520 5530 5540 5550 5560 5570 5580 5590 5600

....|....|....|....|....|....|....|....|....|....|....|....|....|....|....|....|....|....|....|....|

**Col-0**  **TCAAGATCCTTGATCGATATGGGAAACAGCATGCTGATGATCTTAAAGCCTTGGTAATACAAACATTTTGAATCTTTTCCCTGATGGAGTTTTATAAGGC**

**Var2-6** **TCAAGATCCTTGATCGATATGGGAAACAGCATGCTGATGATCTTAAAGCCTTGGTAATACAAACATTTTGAATCTTTTCCCTGATGGAGTTTTATAAGGC**

5610 5620 5630 5640 5650 5660 5670 5680 5690 5700

....|....|....|....|....|....|....|....|....|....|....|....|....|....|....|....|....|....|....|....|

**Col-0**  **GTAAATTTACTATTAGTTTGCCGAGTGATCCTAAATATAAAATGAGGTGGTGGCTCCACATGCATTATGCATACCGCAATTTTCATAGCCCTTGTCTTTT**

**Var2-6** **GTAAATTTACTATTAGTTTGCCGAGTGATCCTAAATATAAAATGAGGTGGTGGCTCCACATGCATTATGCATACCGCAATTTTCATAGCCCTTGTCTTTT**

5710 5720 5730 5740 5750 5760 5770 5780 5790 5800

....|....|....|....|....|....|....|....|....|....|....|....|....|....|....|....|....|....|....|....|

**Col-0**  **ACCGCTTCTTCTGTCCCTTTTTCATGGGCAGGATCATCAGTCAAAAGCTCTGAACTATGGTTCACACTATGAGCTACTTGAACTTGTGGATAGGTTAGTA**

**Var2-6** **ACCGCTTCTTCTGTCCCTTTTTCATGGGCAGGATCATCAGTCAAAAGCTCTGAACTATGGTTCACACTATGAGCTACTTGAACTTGTGGATAGGTTAGTA**

5810 5820 5830 5840 5850 5860 5870 5880 5890 5900

....|....|....|....|....|....|....|....|....|....|....|....|....|....|....|....|....|....|....|....|

**Col-0**  **CTACTAACTAAGACTATATTTGCTCTCCACCTTTGATTACAAAGGAATTAGTTTTTTTTTTGTCAAACTATGAATATATGCAGCAAGCTTGTGGGATCAA**

**Var2-6** **CTACTAACTAAGACTATATTTGCTCTCCACCTTTGATTACAAAGGAATTAGTTTTTTTTTTGTCAAACTATGAATATATGCAGCAAGCTTGTGGGATCAA**

5910 5920 5930 5940 5950 5960 5970 5980 5990 6000

....|....|....|....|....|....|....|....|....|....|....|....|....|....|....|....|....|....|....|....|

**Col-0**  **ATGTCAAAAATGTGAGTATCGATGCTCTTGTTCAACTGGAGGAACACCTTGAGACTGCCCTCTCCGTGACTAGAGCCAAGAAGGTAAGTTGATTTCGTAA**

**Var2-6** **ATGTCAAAAATGTGAGTATCGATGCTCTTGTTCAACTGGAGGAACACCTTGAGACTGCCCTCTCCGTGACTAGAGCCAAGAAGGTAAGTTGATTTCGTAA**

6010 6020 6030 6040 6050 6060 6070 6080 6090 6100

....|....|....|....|....|....|....|....|....|....|....|....|....|....|....|....|....|....|....|....|

**Col-0**  **TGTCTACTCCTTTCTGAATTTTGTTTGCTGAGAACAACCGTGCTGCTTTTGTTTGTTGCAGACCGAACTCATGTTGAAGCTTGTTGAGAATCTTAAAGAA**

**Var2-6** **TGTCTACTCCTTTCTGAATTTTGTTTGCTGAGAACAACCGTGCTGCTTTTGTTTGTTGCAGACCGAACTCATGTTGAAGCTTGTTGAGAATCTTAAAGAA**

6110 6120 6130 6140 6150 6160 6170 6180 6190 6200

....|....|....|....|....|....|....|....|....|....|....|....|....|....|....|....|....|....|....|....|

**Col-0**  **AAGGTCAGATATTTGCTACCAATTTTATTGTACATCAGATATATCCTCTTCTGTGTTGTCTCTGTTACTTTAAGTCTGCTTAACGAGCTTGCACACATAT**

**Var2-6** **AAGGTCAGATATTTGCTACCAATTTTATTGTACATCAGATAGATCCTCTTCTGTGTTGTCTCTGTTACTTTAAGTCTGCTTAACGAGCTTGCACACATAT**

6210 6220 6230 6240 6250 6260 6270 6280 6290 6300

....|....|....|....|....|....|....|....|....|....|....|....|....|....|....|....|....|....|....|....|

**Col-0**  **TTGCAACTTTCTTCATATGTTTTGGATTCCAAATTCTGAAGTTGTTAGGTTTAGAAACTTGATCGGTAATTGCTGAACATTTTGATCTTTAAATCAGGAG**

**Var2-6** **TTGCAACTTTCTTCATATGTTTTGGATTCCAAATTCTGAAGTTGTTAGGTTTAGAAACTTGATCGGTAATTGCTGAACATTTTGATCTTTAAATCAGGAG**

6310 6320 6330 6340 6350 6360 6370 6380 6390 6400

....|....|....|....|....|....|....|....|....|....|....|....|....|....|....|....|....|....|....|....|

**Col-0**  **AAAATGCTGAAAGAAGAGAACCAGGTTTTGGCTAGCCAGGTAACGAAAGCTACATTTCCTAAAAATATATATGCATAACTAATAAGCACTGCGTGTTGTG**

**Var2-6** **AAAATGCTGAAAGAAGAGAACCAGGTTTTGGCTAGCCAGGTAACGAAAGCTACATTTCCTAAAAATATATATGCATAACTAATAAGCACTGCGTGTTGTG**

6410 6420 6430 6440 6450 6460 6470 6480 6490 6500

....|....|....|....|....|....|....|....|....|....|....|....|....|....|....|....|....|....|....|....|

**Col-0**  **TGTCCAATGTCCATGTACATGGACATAGATACACACTCTTATGCTTGCAGATATATATATATATATATATAGTCAGTGCATTTCAATCATTCACTAGTTA**

**Var2-6** **TGTCCAATGTCCATGTACATGGACATAGATACACACTCTTATGCTTGCAGATATATATATATATATATATAGTCAGTGCATTTCAATCATTCACTAGTTA**

6510 6520 6530 6540 6550 6560 6570 6580 6590 6600

....|....|....|....|....|....|....|....|....|....|....|....|....|....|....|....|....|....|....|....|

**Col-0**  **GCACTTTCCTGTCTTGTATAGTTGTATTCTAGACAATTCTTCTCAAGATTAGGGCATTTTGGTTGTTGGTAGTTTGGTTTATTAGGGTTAGTGAGATTAT**

**Var2-6** **GCACTTTCCTGTCTTGTATAGTTGTATTCTAGACAATTCTTCTCAAGATTAGGGCATTTTGGTTGTTGGTAGTTTGGTTTATTAGGGTTAGTGAGATTAT**

6610 6620 6630 6640 6650 6660 6670 6680 6690 6700

....|....|....|....|....|....|....|....|....|....|....|....|....|....|....|....|....|....|....|....|

**Col-0**  **TACTGAATAAGAACAGAATTTTGATAACGGCTGGTTAGAGTTAAGGGAAATCAGATGAAGTTATTTTTTTATTTTTTATCGAGTATAAATTACATGATTG**

**Var2-6** **TACTGAATAAGAACAGAAATTTGATAACGGCTGGTTAGAGTTAAGGGAAATCAGATGAAGTTATTTTTTTATTTTTTATCGAGTATAAATTACATGATTG**

6710 6720 6730 6740 6750 6760 6770 6780 6790 6800

....|....|....|....|....|....|....|....|....|....|....|....|....|....|....|....|....|....|....|....|

**Col-0**  **CTATATCATTTTACTAAATTAAGAAAAAAAAATTCCGGTTGTTGGACATAACTAGGTTTTGGTTCTTCTTCTTCGTTTTTTTCATGTTAAAGTGTTTAAT**

**Var2-6** **CTATATCATTTTACTAAATTAAGAAAAAAAAATTCCGGTTGTTGGACATAACTAGGTTTTGGTTCTTCTTCTTCGTTTTTTTCATGTTAAAGTGTTTAAT**

6810 6820 6830 6840 6850 6860 6870 6880 6890 6900

....|....|....|....|....|....|....|....|....|....|....|....|....|....|....|....|....|....|....|....|

**Col-0**  **TAGGTTTTGGTTCATTTGGAGATTTATGAACCTTTTATAGTCTGGTTAAGTCTGGGTTTGGTAGAGATTCAATAAGATTTCTTGATTCTCTTTCAGGTTA**

**Var2-6** **TAGGTTTTGGTTCATTTGGAGATTTATGAACCTTTTATAGTCTGGTTAAGTCTGGGTTTGGTAGAGATTCAATAAGATTTCTTGATTCTCTTTCAGGTTA**

6910 6920 6930 6940 6950 6960 6970 6980 6990 7000

....|....|....|....|....|....|....|....|....|....|....|....|....|....|....|....|....|....|....|....|

**Col-0**  **TGGTCTGGTTCAGTCTAGTTTAGTTCAATATTGGTTTCCTTGAAGGTTGTGTAAACGTTGTCTATATTTAAGTTAATCACCTTTTAACCAAAAAAAAAAG**

**Var2-6** **TGGTCTGGTTCAGTCTAGTTTAGTTCAATATCGGTTTCCTTGAAGGTTGTGTAAACGTTGTCTATATTTAAGTTAATCACCTTTTAACCAAAAAAAAAAG**

7010 7020 7030 7040 7050 7060 7070 7080 7090 7100

....|....|....|....|....|....|....|....|....|....|....|....|....|....|....|....|....|....|....|....|

**Col-0**  **TTTATGGACCGATTAGTTTTTTTTTTTTTGTTTTTTTTGTTATGGTTAGGTTTGGATCCGAGTGGCTCAGTTCCAACTCCAAGTGTCTAGAAGTAGTGCT**

**Var2-6** **TTTATGGACCGATAAGTTTTTTTTTTTTT-TTTTTTTTGTTATGGTTAGGTTTGGATCCGAGTGGCTCAGTTCCAACTCCAAGTGTCTAGAAGTAGTGCT**

7110 7120 7130 7140 7150 7160 7170 7180 7190 7200

....|....|....|....|....|....|....|....|....|....|....|....|....|....|....|....|....|....|....|....|

**Col-0**  **ACTTTTACATGCTATATATAGGTTAGATTATAAATTATAAACTGGTAAAAGATTATAGATACTGCTTCCAAACTTAAAAGCTTAAACATAAAGAACACAC**

**Var2-6** **ACTTTTACATGCTATATATAGGTTAGATTATAAATTATAAACTGGTTAAAGATTATAGATACTGCTTCCAAACTTAAAAGCTTAAACATAAAGAACACAC**

7210 7220 7230 7240 7250 7260 7270 7280 7290 7300

....|....|....|....|....|....|....|....|....|....|....|....|....|....|....|....|....|....|....|....|

**Col-0**  **AAATTATGAGAAACATAACCTTCTGTAGTGTTTTTTAATGGTTGTTATTTGGTGGTGTGAAAAAGATATTCCTTGGATAGAAGACAAAAAGAGAAAGTGA**

**Var2-6** **AAATTATGAGAAACATAACCTTCTGTAGTGTTTTTTAATGGTTGTTATTTGGTGGTGTGAAAAAGATATTCCTTGGATAGAAGACAAAAAGAGAAAGTGA**

7310 7320 7330 7340 7350 7360 7370 7380 7390 7400

....|....|....|....|....|....|....|....|....|....|....|....|....|....|....|....|....|....|....|....|

**Col-0**  **ATAGTGATTTTGACCTATGATTATCGTACAGATGGAGAATAATCATCATGTGGGAGCAGAAGCTGAGATGGAGATGTCACCTGCTGGACAAATCTCCGAC**

**Var2-6** **ATAGTGATTTTGACCTATGATTATCGTACAGATGGAGAATAATCATCATGTAGGAGCAGAAGCTGAGATGGAGATGTCACCTGCTGGACAAATCTCCGAC**

Synonymous polymorphism on *FLC* exon 7

7410 7420 7430 7440 7450 7460 7470 7480 7490 7500

....|....|....|....|....|....|....|....|....|....|....|....|....|....|....|....|....|....|....|....|

**Col-0**  **AATCTTCCGGTGACTCTCCCACTACTTAATTAGCCACCTTAAATCGGCGGTTGAAATCAAAATCCAAAACATATATAATTATGAAGAA--AAAAAAAATA**

**Var2-6** **AATCTTCCGGTGACTCTCCCACTACTTAATTAGCCACCTTAAATCGGCGGTTGAAATCAAAATCCAAAACATATATAATTATGAAGAAGAAAAAAAAATA**

7510 7520 7530 7540 7550 7560 7570 7580 7590 7600

....|....|....|....|....|....|....|....|....|....|....|....|....|....|....|....|....|....|....|....|

**Col-0**  **AGATATGTAATTATTCCGCTGATAAGGGCGAGCGTTTGTATATCTTAATACTCTCTCTTTGGCCAAGAGACTTTGTGTGTGATACTTAAGTAGACGGAAC**

**Var2-6** **AGATATGTAATTATTCCGCTGATAAGGGCGAGCGTTTGTATATCTTAATACTCTCTCTTTGGCCAAGAGACTTTGTGTGTGATACTTAAGTAGACGGAAC**

7610 7620 7630 7640 7650 7660 7670 7680 7690 7700

....|....|....|....|....|....|....|....|....|....|....|....|....|....|....|....|....|....|....|....|

**Col-0**  **TAAGTCAATACTATCTGTTTTAAGACAAAAGGTTGATGAACTTTGTACCTTATTCGTGTGAGAATTGCATCGAGATCTTGAGTGTATGTGTTCTTCACTT**

**Var2-6** **TAAGTCAATACTATCTGTTTTAAGACAAAAGGTTGATGAACTTTGTACCTTATTCGTGTGAGAATTGCATCGAGATCTTGAGTGTATGTGTTCTTCTCTT**

7710 7720 7730 7740 7750 7760 7770 7780 7790 7800

....|....|....|....|....|....|....|....|....|....|....|....|....|....|....|....|....|....|....|....|

**Col-0**  **CTGTCAAAAACTTGTGTTTGCTTCACAGTGAAGAAGCCTACGGCTTATTTTGCAACAGGGACGTGGCTCTCTCTCTCTCTCT--GCGCGTTTTTTCTCTC**

**Var2-6** **CTGTCAAAAACTTGTGTTTGCTTCACAGTGAAGAAGCCTACGGCTTATTTTGCAACAGGGACGTGGCTCTCTCTCTCTCTCTCTGCGCGTTTTTTCTCTC**

7810 7820 7830 7840 7850 7860 7870 7880 7890 7900

....|....|....|....|....|....|....|....|....|....|....|....|....|....|....|....|....|....|....|....|

**Col-0**  **GTCGTAATTAATTTGTTTTTATCCTAAACGCGTATGGTTGGCATGGGTTTTTTGGGCCTATGTCGGTCACATTCGGCCCAATAAGCTTAGTTCTTAAAGT**

**Var2-6** **GTCGTAATTAATTTGTTTTTATCCTAAACGCGTATGGTTGGCATGGGTTTTTTGGGCCTATGTCGGTCACATTCGGCCCAATAAGCTTAGTTCTTAAAGT**

7910 7920 7930 7940 7950 7960 7970 7980 7990 8000

....|....|....|....|....|....|....|....|....|....|....|....|....|....|....|....|....|....|....|....|

**Col-0**  **CATTGCTGTTAACGTGACCGACCTCTTCCATATTTTCTTTGGACTAAATTCACTTCCGTAGTTCCGTCATCCATGAAGGATCCCTTAAACTAATCCACAT**

**Var2-6** **CATTGCTGTTAACGTGACCGACCTCTTCCATATTTTCTTTGGACTAAATTCACTTCCGTAGTTCCGTCATCCATGAAGGATCCCTTAAACTAATCCACAT**

8010 8020 8030 8040 8050 8060 8070 8080 8090 8100

....|....|....|....|....|....|....|....|....|....|....|....|....|....|....|....|....|....|....|....|

**Col-0**  **TTATATAATGATAACACTTCTTTTTTTCCCACTAAAATGATAATACTTTTAGACATTATATGTATAAATATAATTCACATATAGAATAGAAAAAGTTACG**

**Var2-6** **TTATATAATGATAACACTTCTTTTTT-CCCACTAAAATGATAATACTTTTAGACATTATATGTATAAATATAATTCACATATAGAATAGAAAAAGTTACG**

8110 8120 8130 8140 8150 8160 8170 8180 8190 8200

....|....|....|....|....|....|....|....|....|....|....|....|....|....|....|....|....|....|....|....|

**Col-0**  **CTCGTATTTATATTAATTTAGATCAACAGTTCCAGACGCCATTGTCATTAATCACCTTTTAACATTTCTGATTTAAATTAAATCATAGTCTATATACTCT**

**Var2-6** **CTCGTATTTATATTAATTTAGATCAACAGTTCCAGACGCCATTGTCATTAATCACCTTTTAACATTTCTGATTTAAATTAAATCATAGTCTATATACTCT**

8210 8220 8230 8240 8250 8260 8270 8280 8290 8300

....|....|....|....|....|....|....|....|....|....|....|....|....|....|....|....|....|....|....|....|

**Col-0**  **GATTCAGCCTACAAGATGATAAATAGAGAATCGTCAGTGTTAAAATGCACTCTTACGTAACGAGGAAACATAAGCACGTTCTGGTCGGACCGAAGCTAAC**

**Var2-6** **GATTCAGCCTACAAGATGATAAATAGAGAATCGTCAGTGTTAAAATGCACTCTTACGTAACGAGGAAACATAAGCACGTTCTGGTCGGACCGAAGCTAAC**

8310 8320 8330 8340 8350 8360 8370 8380 8390 8400

....|....|....|....|....|....|....|....|....|....|....|....|....|....|....|....|....|....|....|....|

**Col-0**  **CAGCAATTTCATCACTCTATTTCACATTCTAGTTAATCCACAAAATTTTGATATACAAAATTATAAT-ATTATTCATTGTATAATTTAAAAGTGAAAGGT**

**Var2-6** **CAGCAATTTCATCACTCTATTTCACATTCTAGTTAATCCACAAAATTTTGATATACAAAATTATAATTATTATTCATTGTATAATTTAAAAGTGAAAGGT**

8410 8420 8430 8440 8450 8460 8470 8480 8490 8500

....|....|....|....|....|....|....|....|....|....|....|....|....|....|....|....|....|....|....|....|

**Col-0**  **TTGTTAAATATAGTAGTAGAATTACAATTTAATTACCAACATAAAGTCAAATGCCGAATAAAGCATTCGAAATAAAATTGTAAAGTCCGATGGAGACGTA**

**Var2-6** **TTGTTAAATATAGTAGTAGAATTACAATTTAATTACCAACATAAAGTCAAATGCCGAATAAAGTATTCGAAATAAAATTGTAAAGTCCGATGGAGACGTA**

8510 8520 8530 8540 8550 8560 8570 8580 8590 8600

....|....|....|....|....|....|....|....|....|....|....|....|....|....|....|....|....|....|....|....|

**Col-0**  **GACTAGAAATAACAAGTACCATACGAGTTGACTTGTGTAGAAGGCAACACAAACTTTCTCGCCGAGTACGGCGGAGGAGCAGCCGCAAGTGGATCCAGCC**

**Var2-6** **GACTAGAAATAACAAGTACCATACGAGTTGACTTGTGTAGAAGGCAACACAAACTTTCTCGCCGAGTACGGCGGAGGAGCAGCCGCAAGTGGATCCAGCC**

8610 8620 8630 8640 8650 8660 8670 8680 8690 8700

....|....|....|....|....|....|....|....|....|....|....|....|....|....|....|....|....|....|....|....|

**Col-0**  **GACACTAAGGAAGCTTTTAATTTGCACAAGATGCGATACACTTACACCTGACCCTTCAATTTCCTACTTATATAATACATAGTTTCAGTTTTCACATAAT**

**Var2-6** **GACACTAAGGAAGCTTTTAATTTGCACAAGATGCGATACACTTACACCTC-CCCTTCAATTTCCTACTTATATAATACATAGTTTCAGTTTTCACATAAT**

8710 8720 8730 8740 8750 8760 8770

....|....|....|....|....|....|....|....|....|....|....|....|....|....|....|..

**Col-0**  **ACACAAAACCCTTTCACTAATAACAATCTCCAAATGGCTAAATCATTTGTACCTCTCATCGCTGTGTTATGCGTTT**

**Var2-6** **ACACAAAACCCTTTCACTAATAACAATCTCCAAATGGCTAAATCATTTGCACCTCTCATCGCTGTGTTATGCGTTT**
